# Dual COX-2/TNF-α Inhibitors as Promising Anti-inflammatory and Cancer Chemopreventive Agents: A Review

**DOI:** 10.5812/ijpr-151312

**Published:** 2024-10-29

**Authors:** Mobina Tajdari, Amirreza Peyrovinasab, Maryam Bayanati, Mohammad Ismail Mahboubi Rabbani, Amir Hossein Abdolghaffari, Afshin Zarghi

**Affiliations:** 1 Department of Pharmacology and Toxicology, Faculty of Pharmacy, Tehran Medical Sciences, Islamic Azad University, Tehran, Iran; 2 GI Pharmacology Interest Group (GPIG), Universal Scientific Education and Research Network (USERN), Tehran, Iran; 3 Department of Food Technology Research, National Nutrition, and Food Technology Research Institute, Faculty of Nutrition Sciences and Food Technology, Shahid Beheshti University of Medical Sciences, Tehran, Iran; 4 Department of Medicinal Chemistry, Faculty of Pharmacy, Tehran Medical Sciences, Islamic Azad University, Tehran, Iran; 5 Department of Medicinal Chemistry, School of Pharmacy, Shahid Beheshti University of Medical Sciences, Tehran, Iran

**Keywords:** COX-2, TNF-α, Dual Inhibitors, Cancer

## Abstract

Cyclooxygenases (COX) play a pivotal role in inflammation and are responsible for the production of prostaglandins (PGs). Two types of COXs have been identified as key biological targets for drug design: Constitutive COX-1 and inducible COX-2. Nonsteroidal anti-inflammatory drugs (NSAIDs) target COX-1, while selective COX-2 inhibitors are designed for COX-2. These COX isoforms are involved in multiple physiological and pathological pathways throughout the body. Overproduction of tumor necrosis factor-alpha (TNF-α) plays a role in COX-2's inflammatory activity. Tumor necrosis factor-alpha can contribute to cardiac fibrosis, heart failure, and various cancers by upregulating the COX-2/PGE2 axis. Therefore, suppressing COX activity has emerged as a potentially effective treatment for chronic inflammatory disorders and cancer. This review explores the mechanisms of TNF-α-induced COX-2/PGE2 expression, a significant pathophysiological feature of cancer development. Furthermore, we summarize chemical compounds with dual COX-2/TNF-α inhibitory actions, providing an overview of their structure-activity relationship. These insights may contribute to the development of new generations of dual-acting COX-2/TNF-α inhibitors with enhanced efficacy.

## 1. Introduction

Inflammatory processes play a crucial role in various diseases, including autoimmune disorders, cardiovascular disease, and cancer. Overexpression of cyclooxygenase-2 (COX-2) and tumor necrosis factor-alpha (TNF-α) has been associated with pathological conditions (1). Certain types of lipids, such as those found in arachidonic acid (AA), linoleic acid, eicosapentaenoic acid, and docosahexaenoic acid commonly found in fish and vegetable oils can be converted by the body into pro-inflammatory fats. Arachidonic acid, a 20-carbon polyunsaturated fatty acid, is typically esterified to the second carbon in membrane phospholipids, where it gives rise to a variety of lipid compounds known as eicosanoids (2). Eicosanoids are specialized lipids derived from 20-carbon fatty acids like AA, produced in many cells through specific biochemical pathways (3). These molecules function as lipid-based secondary messengers in cell signaling, comparable to other chemical messengers such as calcium ions, inositol trisphosphate, cyclic nucleotides, and diacylglycerol (4). Eicosanoids can be released into the extracellular space, penetrate cell membranes, and activate receptors in nearby cells, triggering localized responses within small tissue regions (5). These lipid signaling molecules are critical in regulating inflammation and carcinogenesis and are generated by enzymes, such as COX, lipoxygenase (LOX), and cytochrome P450 (6).

Phospholipases, including calcium-dependent cytosolic phospholipase A2, are responsible for releasing AA from phospholipids in the outer layer of the nuclear membrane. Once released, free fatty acids can be converted by COX and LOX enzymes. This process generates 4-series leukotrienes and hydroxy eicosatetraenoic acids via the LOX pathway, and 2-series prostaglandins (PGs) and thromboxanes via the COX pathway (7). This leads to the formation of various oxylipin metabolites with distinct structures and effects on the body (8). Eicosanoid signaling, similar to the cytokine signaling pathway and inflammasome formation, has traditionally been viewed as part of the body's response to infection and injury, activating the immune system and causing oxidative stress (9). However, new research has revealed that certain eicosanoids and related lipids also play a role in reducing inflammation and promoting healing (10).

**Figure 1.**
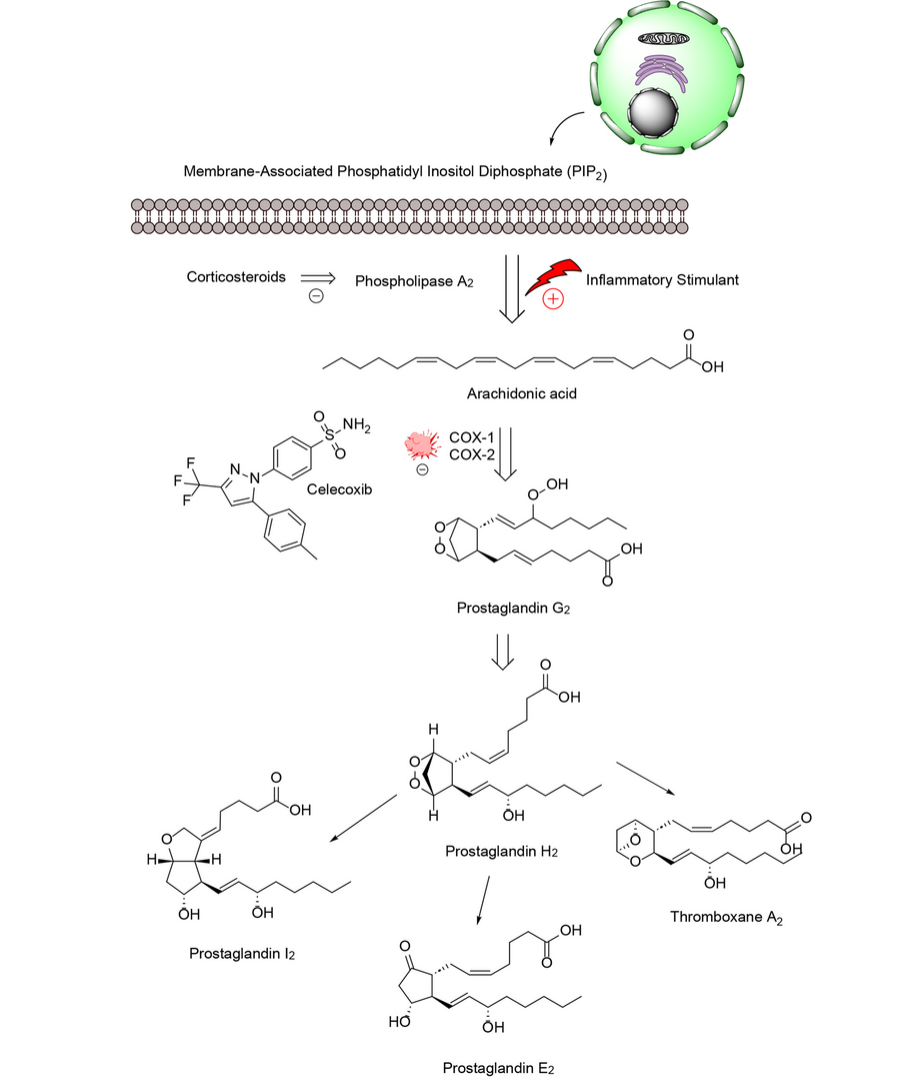
The prostanoids pathway

Eicosanoids (Figure 1) are involved in both promoting inflammation and contributing to the development of inflammation-associated cancers (11, 12). Inflammation-inducing chemicals produced by tumor cells and the surrounding tissue can influence tumor growth in multiple ways (13). Specifically, AA produces specialized compounds that contribute to inflammation and cell proliferation in this context (14). Pro-inflammatory eicosanoids are produced in large quantities by nearby cells and various types of cancerous cells. These biologically active lipid compounds can influence tumor growth through mechanisms such as activating receptors on malignant epithelial cells, modulating cell proliferation, apoptosis, migration, and invasion, and inducing growth factors, inflammatory cytokines, and angiogenic agents. These processes can promote tumor progression, tumor-associated angiogenesis, and resistance to immune responses (15).

Tumor necrosis factor-alpha is a cytokine produced by various cell types, including macrophages and T-cells, playing a crucial role in inflammation, immune responses, and cell death. Elevated levels of TNF-α are associated with numerous diseases, such as rheumatoid arthritis, inflammatory bowel disease, and cancer (16). Dual COX-2/TNF-α inhibitors exert their therapeutic effects through multiple mechanisms. By inhibiting COX-2, these agents reduce the production of PGs, leading to a decrease in inflammation, pain, and fever (17). Additionally, dual inhibitors can lower inflammation and promote tissue healing by directly inhibiting or neutralizing TNF-α production and activity (18). These inhibitors may also modulate other inflammatory pathways, such as the NF-κB pathway, further contributing to their anti-inflammatory effects (19).

Due to these combined actions, dual COX-2/TNF-α inhibitors have been investigated as chemopreventive agents and as adjuvants in cancer therapy. They have the potential to inhibit tumor growth, angiogenesis, and metastasis (20). This review will explore the pharmacological properties, mechanisms of action, and structure-activity relationships (SAR) of these compounds as anti-inflammatory and cancer chemopreventive agents. Understanding the benefits and limitations of dual COX-2/TNF-α inhibition could help enhance patient outcomes and lead to the development of new therapeutic strategies.

## 2. Cyclooxygenases-2 Roles in Cancer Pathophysiology

**Figure 2.**
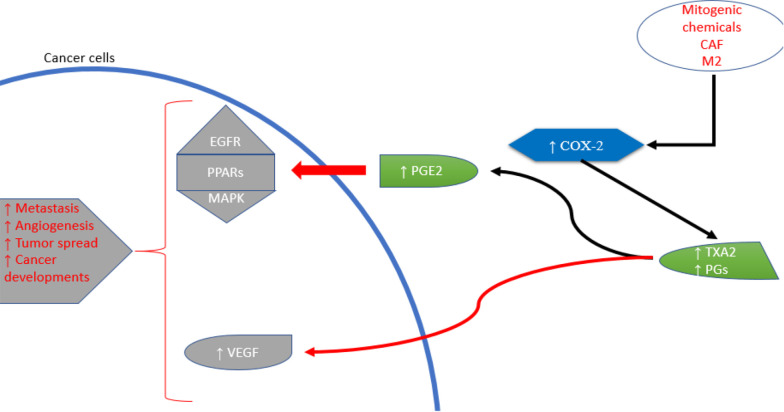
Cyclooxygenases-2 (COX-2) roles in cancer development

Cyclooxygenases-2 is an enzyme produced in the body during conditions such as inflammation and cancer. It contributes to the production of substances known as prostanoids, which include both PGs and thromboxanes (Figure 2) (21). Various stimuli, such as pro-inflammatory cytokines, growth factors, mitogens, oncogenes, and hypoxia, can increase COX-2 expression (22). The role of COX-2 in the initiation, progression, and metastasis of cancers is facilitated through several mechanisms. Due to their peroxidase activity, COX enzymes can produce carcinogenic and mutagenic compounds (23). The prostaglandin E2 (PGE2) signaling pathway involves nuclear receptor pathways (PPARs), the Ras-mitogen-activated protein kinase (MAPK) pathway, and the epidermal growth factor receptor (EGFR) pathway. These cellular processes promote cancer development and growth. Prostaglandin E2’s downstream targets include angiogenic factors, anti-apoptotic factors, chemokines and their receptors, and immunosuppressive molecules (24).

Cancer cells, specific immune cells such as macrophages (type 2), and cancer-associated fibroblasts (CAFs) release COX-2 into the tumor microenvironment (25). Research has explored the involvement of COXs, especially COX-2, and PGs in cancer-related inflammation (26). Cyclooxygenases-2 plays a crucial role in neoplastic transformation, cancer progression, inhibition of apoptosis, and promotion of angiogenesis, proliferation, and tumor growth. Thus, COX-2 inhibitors, known as coxibs, show potential for cancer prevention and treatment (27). Cyclooxygenases-2 overexpression has been implicated in various cancers, including lung, pancreatic, and gastric cancers, among others (28). Single nucleotide variants in the COX-2 gene have been associated with increased susceptibility to esophageal cancer through genetic variations (29). In cultured adult breast cancer cells, COX inhibitors were found to reduce aromatase mRNA expression and enzyme activity in a dose-dependent manner, suggesting potential applications in hormone-dependent breast cancer therapies (30). In gastric carcinoma, elevated COX-2 expression was correlated with higher levels of vascular endothelial growth factor (VEGF)-C and lymphatic spread, highlighting COX-2’s key role in lymphatic metastasis and the VEGF-C lymphangiogenic pathway (31). Since COX-2 produces PGs, overexpression of COX-2 can lead to excessive PG production, which may promote cancer growth by directly stimulating cellular proliferation (32).

It has been previously explained that cluster of differentiation 133 (CD133), also known as prominin-1, a marker for stem cells, is associated with COX-2 expression. Cells expressing CD133 show higher levels of COX-2 compared to those without CD133 (33). Celecoxib, a COX-2 inhibitor, reduced the radiation resistance of CD133-positive cells by increasing cell division control protein 2 (cdc2) activity (34). The COX-2-PGE2-EPs pathway plays a crucial role in inflammation leading to gynecological cancers (35). It was shown that fibronectin, a binding agent for α5β1 integrin, promotes the development of pulmonary cancer cells through α5β1-dependent signaling. NS398 and nimesulide, both selective COX-2 inhibitors, reduced mRNA expression and protein synthesis of the integrin α5 subunit, which was associated with decreased adherence of non-small cell lung cancer cells to fibronectin. This effect was linked to the transcriptional regulation of the integrin α5 gene, driven by Erk activation, increased Sp1 and decreased AP-1 binding to DNA, and inhibition of stress-activated protein kinase/c-Jun N-terminal kinase signaling, resulting in reduced α5β1 integrin expression in non-small cell lung cancer cells (36).

## 3. Tumor Necrosis Factor-Alpha Role in Cancer Pathophysiology

**Figure 3.**
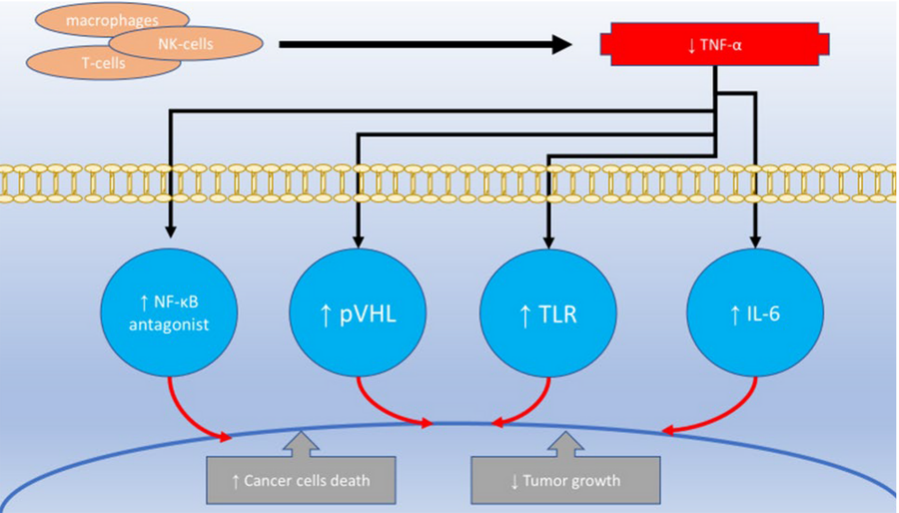
Tumor necrosis factor-alpha (TNF-α) roles in cancer development

Tumor necrosis factor-alpha is a protein that belongs to the TNF/Tumor Necrosis Factor Receptor (TNFR) family (Figure 3). It is a type of protein that is attached to the outer cell membrane (37). Activated macrophages, T-lymphocytes, and natural killer cells are the primary producers of TNF-α. Other cell types, such as cancerous cells, smooth muscle cells, and fibroblasts, also exhibit lower levels of TNF-α transcription (38). Tumor necrosis factor-alpha plays a significant role in regulating cell survival and inflammation within the body (39). When TNF-α was first identified in 1975, it was observed to cause tumor necrosis and hemorrhage, leading to its classification as a highly effective anticancer cytokine (40). It was later discovered that administering high doses of TNF-α directly to cancer cells could induce cell death, although this effect typically occurs in combination with other growth-inhibiting antimetabolites (41, 42). In addition, liposomal encapsulation of TNF-α has been shown to prolong its distribution time and increase therapeutic TNF-α levels in tumors when combined with anticancer drugs in systemic treatments in rat models (43).

Modest quantities of TNF-α seem to increase the permeability of cancerous blood vessels, enhancing the tissue penetration of chemotherapy drugs and disrupting tumor angiogenesis (44). However, this powerful cytokine also has a downside. Despite its name, TNF-α is found in diseases such as chronic inflammation, autoimmunity, and cancer, particularly in middle-aged and older individuals (45). Research has demonstrated that TNF-α plays a crucial role in the direct lysis of carcinoma cells in leukemia cell lines and enhances the anti-tumor effects of various chemotherapy drugs on leukemia and lymphoma cell lines (46). It has also been shown that TNF-α promotes the growth of myeloma cells and contributes to myeloma bone disease, both directly and through the activation of interleukin 6 (IL-6) (47). Toll-like receptor signaling may be involved in the production of TNF-α in cancer (48). Tumor necrosis factor-alpha in the body is linked to increased expression of the chemokine receptor CXCR4 and its ligand CXCL12 in cancer cells, both in lab-cultured cells and patient-derived samples (49). Additionally, inflammation-inducing cytokines like TNF-α and IL-6 play a critical role in causing anemia in cancer patients, not only by promoting inflammation but also by influencing genes such as GATA-1 and GATA-2, which regulate red blood cell production (50).

Among the factors that contribute to inflammation, TNF-α is believed to have a strong effect when combined with interferon-gamma (IFN-γ). This combination helps mesenchymal stem cells reduce inflammation and makes them less likely to trigger an immune response (51). Additionally, a novel connection has been identified between the TNF-α gene and kidney cancer. The tumor suppressor gene, Von Hippel-Lindau (pVHL), was found to block TNF-α, suggesting a role in preventing tumor growth (52). Generally, high levels of certain cytokines in the body, such as IL-1, IL-6, and TNF-α, can cause symptoms like fevers, night sweats, and weight loss (53). Tumor necrosis factor blockers prevent the release of cytokines and chemokines, reducing inflammation, angiogenesis, and the breakdown of the extracellular matrix in individuals with chronic inflammatory conditions (54). Regarding cancer treatment, two key effects of TNF blockers on the immune system in patients with inflammatory diseases are noteworthy: they modulate TReg cell activity and reduce T-helper cell inflammatory responses by decreasing IL-17 production (55). Furthermore, targeting NF-κB antagonists and upregulating IκB, an NF-κB inhibitor, has been shown to enhance TNF-α-induced cell death in cancer cells (56).

## 4. Connections Between Cyclooxygenases-2 and Tumor Necrosis Factor-Alpha

**Figure 4.**
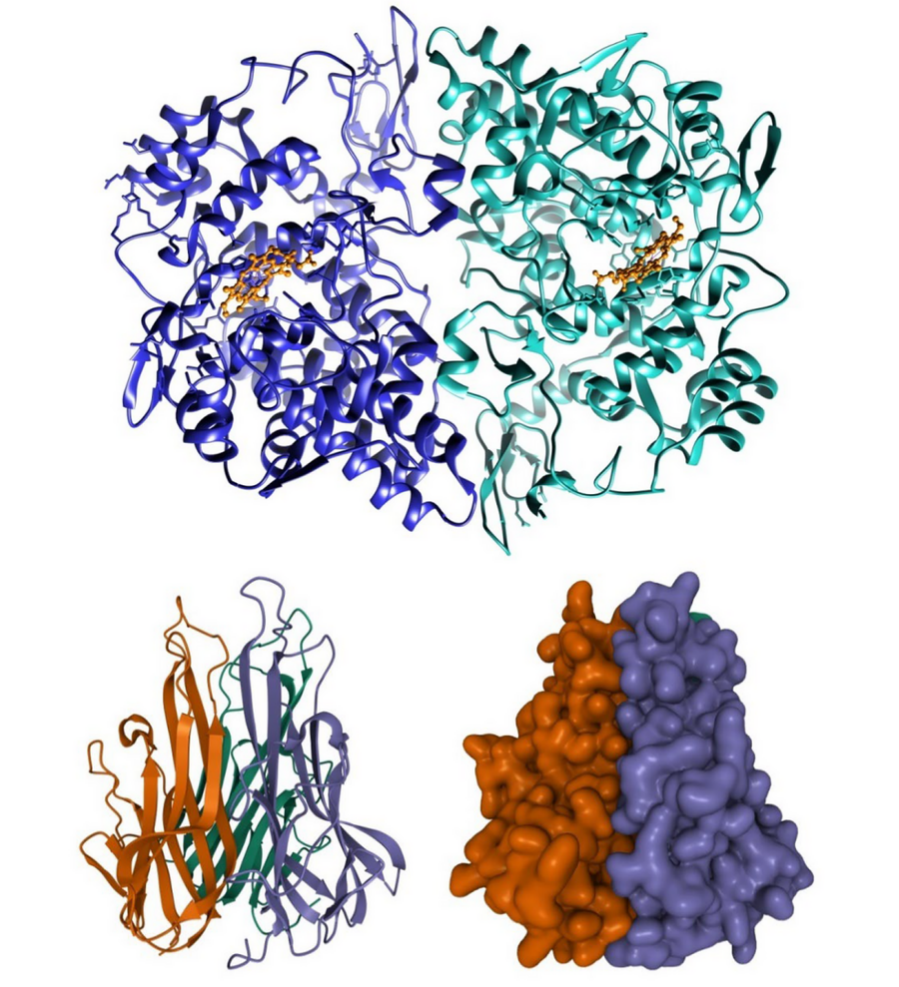
Three-dimensional structures of cyclooxygenases-2 (COX-2) (PDB ID: 1CX2) and tumor necrosis factor-alpha (TNF-α) (PDB ID: 1TNF)

Activated microglia can produce pro-inflammatory cytokines, such as TNF-α, along with potentially neurotoxic components like nitric oxide (NO) and PGs, synthesized through inducible nitric oxide synthase (iNOS) and COX-2, respectively (57). Tumor necrosis factor-alpha can also activate other inflammation-causing pathways, such as those regulated by COX-2 (58). Tumor necrosis factor-alpha, a potent pro-inflammatory cytokine, significantly promotes the production of COX-2 and PGE2 in fibroblasts associated with colon cancer (59). Both TNF-α and IL-1β increase COX-2 and PGE2 production (60). Tumor necrosis factor-alpha has been shown to significantly elevate PGE2 synthesis and COX-2 transcription in carcinoma-associated colon fibroblasts (Figure 4). In vitro experiments demonstrated that the proliferation and aggressiveness of colon cancer cells increased when stromal COX-2 signaling was activated (59). Conversely, COX-2, which produces PGs and thromboxanes, contributes to inflammation and healing, particularly in inflammatory diseases like ulcerative colitis. This may hinder the effectiveness of TNF-α inhibitors (61). Tumor necrosis factor-induced stimulation of primary monocytes in responders has been associated with dual COX-2 (PTGS2) expression (62). Additionally, researchers have found that the NF-κB signaling pathway may stimulate the production of COX-2 and TNF-α in macrophages (63, 64). In human periodontal fibroblasts, the NF-κB transcription factor plays a key role in regulating COX-2 expression during tyrosine kinase-mediated PGE2 synthesis induced by TNF-α (65).

Products of the 5-LOX pathway (leukotrienes) are particularly important in inflammatory conditions. Leukotrienes increase microvascular permeability and act as powerful chemotactic agents. Inhibiting 5-LOX also indirectly reduces TNF-α transcription, a crucial cytokine in inflammation. This has led to efforts to develop drugs that inhibit both 5-LOX and COX, known as dual-acting anti-inflammatory drugs (66).

Celecoxib, a COX-2 inhibitor, also suppressed the NF-κB-regulated COX-2 expression in patients with non-small cell lung cancer, and this inhibition was associated with a reduction in TNF-α-induced COX-2 transcription (67). The activation of NF-κB has been shown to induce the expression of inflammatory cytokines, such as TNF-α, and adhesion molecules, which require reactive oxygen species produced by the 5-LOX family (68). This indicates that p38 MAPK and NF-κB regulate COX-2 production in response to certain inflammatory signals, including TNF-α, IL-1β, and IFN-γ (69). Cyclooxygenases-2 antagonists may increase the susceptibility of cancer cells to TNF-α-induced cell death by inducing the production of reactive oxygen species or inhibiting the NF-κB pathway (70).

## 5. Structure-Activity Relationship of Cyclooxygenases-2 Inhibitors

Recent studies have led to the development of an extensive group of selective COX-2 inhibitors, some of which have shown potent TNF-α regulatory activity (71-94). The side effects associated with well-known COX-2 inhibitors prompted scientists to create novel compounds to mitigate these effects. Cyclooxygenases-2 inhibitors are categorized into diaryl heterocycles and non-diaryl heterocycles, with diaryl heterocycles featuring a five-membered core scaffold representing the most selective COX-2 inhibitors. Certain structural features allow for specific COX-2 inhibition, according to medicinal chemists.

In recent years, several compounds have been synthesized containing polar groups and heterocyclic rings, which function as selective inhibitors with low IC_50_ values. Heterocyclic compounds play a crucial role in drug development as they are associated with various organic forms. Coxibs, in particular, possess structural components that serve as novel and intriguing scaffolds in the search for new selective COX-2 inhibitors. Celecoxib has been modified to produce new analogs with fewer adverse effects, leading to the discovery of novel COX-2 inhibitors equipped with thromboxane receptor antagonistic properties.

**Figure 5.**
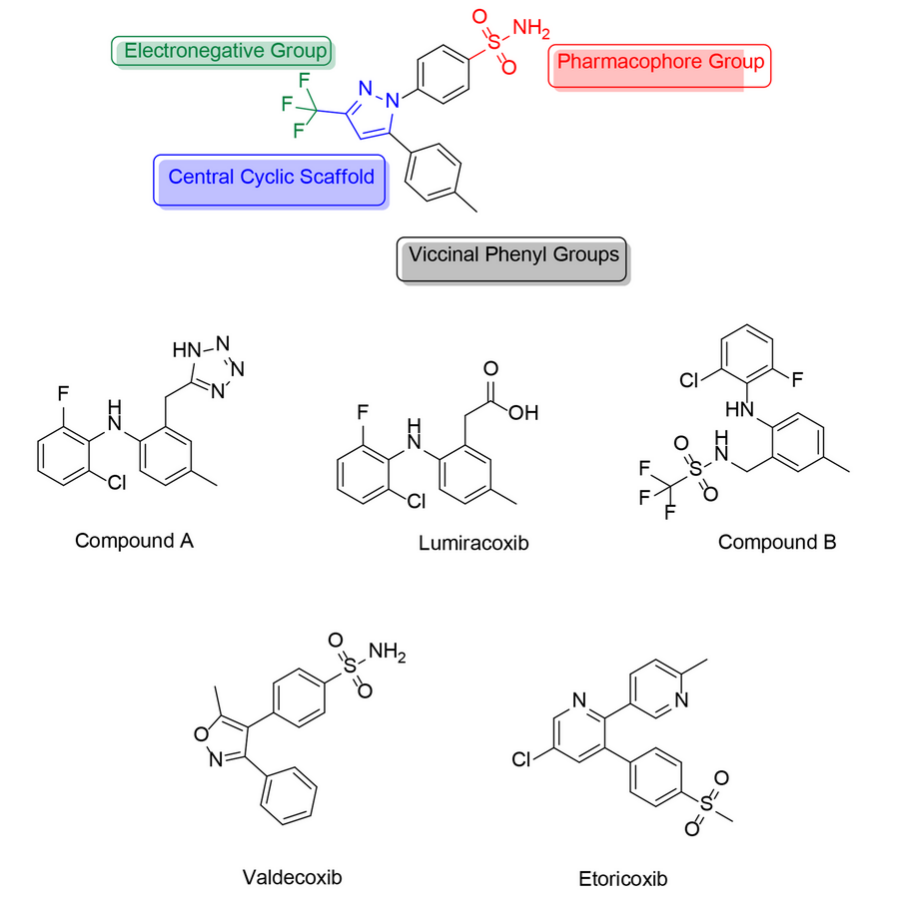
The chemical structures of some prominent prototype cyclooxygenases-2 (COX-2) inhibitors

However, a diaryl heterocycle is not always necessary for a selective COX-2 inhibitor. For example, lumiracoxib (Figure 5) and some of its analogs (compound A, Figure 5) have demonstrated good and selective COX-2 inhibition without including a diaryl heterocycle. In these molecules, the tetrazole ring serves as the scaffold, and no “–SO2CH3/–SO2NH2” pharmacophore group is present. In contrast, this pharmacophore group is found in another analog of lumiracoxib (compound B, Figure 5). Lumiracoxib and valdecoxib (Figure 5) were used as examples to develop new and improved selective COX-2 inhibitors. Using in situ library examination, several structures related to lumiracoxib were discovered, sharing a minimum of two oxygen atoms, two aryl rings, a carboxyl group, and a hydroxyl or amino group.

Halogen, nitrogen, methoxide, alkene, and amide groups are also present, though not uniformly distributed. In contrast, valdecoxib, which has a comparable sulphonamide-based structure, contains two adjacent aromatic moieties, at least two oxygen atoms, a sulphonamide group, and a nitrogen atom. Additional substituents, such as the oxadiazole group, chlorine atom, and benzimidazole ring, are also present but not consistently found across all structures.

Developing new drugs that target COX-2 with fewer side effects remains a highly successful strategy for managing inflammatory diseases. This article reviews the categorization and pharmacological effects of novel COX-2 inhibitors as potential cancer chemopreventive agents. New compounds with significant dual COX-2/TNF-α inhibitory action are highlighted, making them promising candidates for drug development and clinical application.

## 6. Review of Chemical Structures

Aryl propionic acid analogs form a major class of chemical compounds that, due to their ease of interaction with biological systems, are integral to a wide range of physiologically valuable molecules. Several aryl propionic acid analogs have been explored for their COX-2 inhibitory activities. One well-known example is ibuprofen. In 2023, a novel series of ibuprofen derivatives was developed as selective COX-2 inhibitors. To assess the potential anti-inflammatory activity of these newly developed analogs, levels of TNF-α, PGE2, and IL-6 were evaluated. Additionally, a histopathological study was conducted (95).

**Figure 6.**
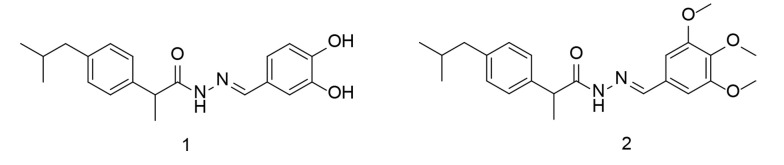
Ibuprofen analogs as selective cyclooxygenases-2 (COX-2) inhibitors

Based on the fact that the carboxyl moiety of ibuprofen binds to Arg-120 and Tyr-355 without extending into the side pocket, it was hypothesized that making the carboxyl group longer by adding larger, more rigid groups with hydrogen-bonding functions could enhance the selectivity of ibuprofen while maintaining its ability to reduce pain and inflammation. Therefore, the new structures were designed by elongating the carboxylate chain and incorporating bulky hydrophobic groups. Compounds 1 and 2 showed the most potent COX-2 activity and selectivity (Figure 6). Both compounds inhibited PGE2 synthesis and lowered rat serum concentrations of IL-6 and TNF-α. Notably, despite their strong anti-inflammatory activity, no adverse effects were observed on the stomach mucosa. Docking simulation studies of the two compounds further supported their increased selectivity for COX-2.

Heterocycles are an essential group of compounds and play a significant role in many drugs that affect biological systems. Various ring-shaped molecules have been studied for their potential to inhibit COX-2. Tetrazole, a widely used heterocyclic core structure, serves as a frequent scaffold in drug design, often replacing carboxyl groups due to its resistance to metabolism. In a study conducted by Labib et al., four innovative series of tetrazole analogs were developed by bioisosteric substitution of the sulfonamide moiety in celecoxib (Figure 5) with tetrazole, incorporating various core structures such as chalcone, isoxazole, and pyrazole (96). These compounds demonstrated greater specificity for COX-2 than traditional drugs like indomethacin and celecoxib. The tested compounds exhibited potent COX-2 inhibitory activity in vitro. Notably, the trimethoxy analogs showed improved COX-2 Selectivity Index (SI) values, surpassing that of celecoxib.

**Figure 7.**
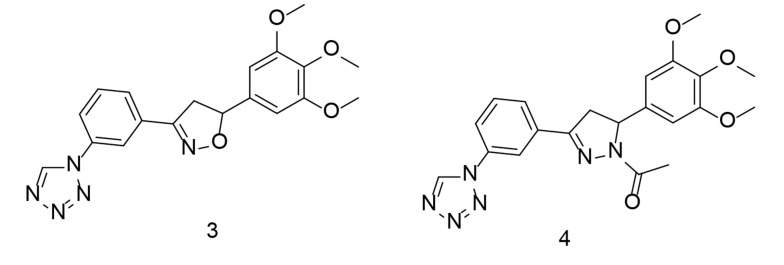
Tetrazole analogs as dual cyclooxygenases-2 (COX-2)/tumor necrosis factor-alpha (TNF-α) inhibitors

The compounds with the highest efficacy were evaluated for their anti-inflammatory activity and effects on plasma levels of TNF-α, PGE2, and IL-6. Among the newly synthesized analogs, compounds 3 and 4 (Figure 7) demonstrated superior anti-inflammatory properties and caused fewer gastric ulcers compared to celecoxib. Additionally, they effectively inhibited PGE2 production to a greater extent than celecoxib. Compound 4 significantly reduced IL-6 and TNF-α levels in the blood of treated rats, showing comparable or even better performance than celecoxib. Docking studies revealed that the most active compounds formed strong binding interactions, and the overall energy scores supported the impressive COX-2 inhibitory activity observed.

**Figure 8.**
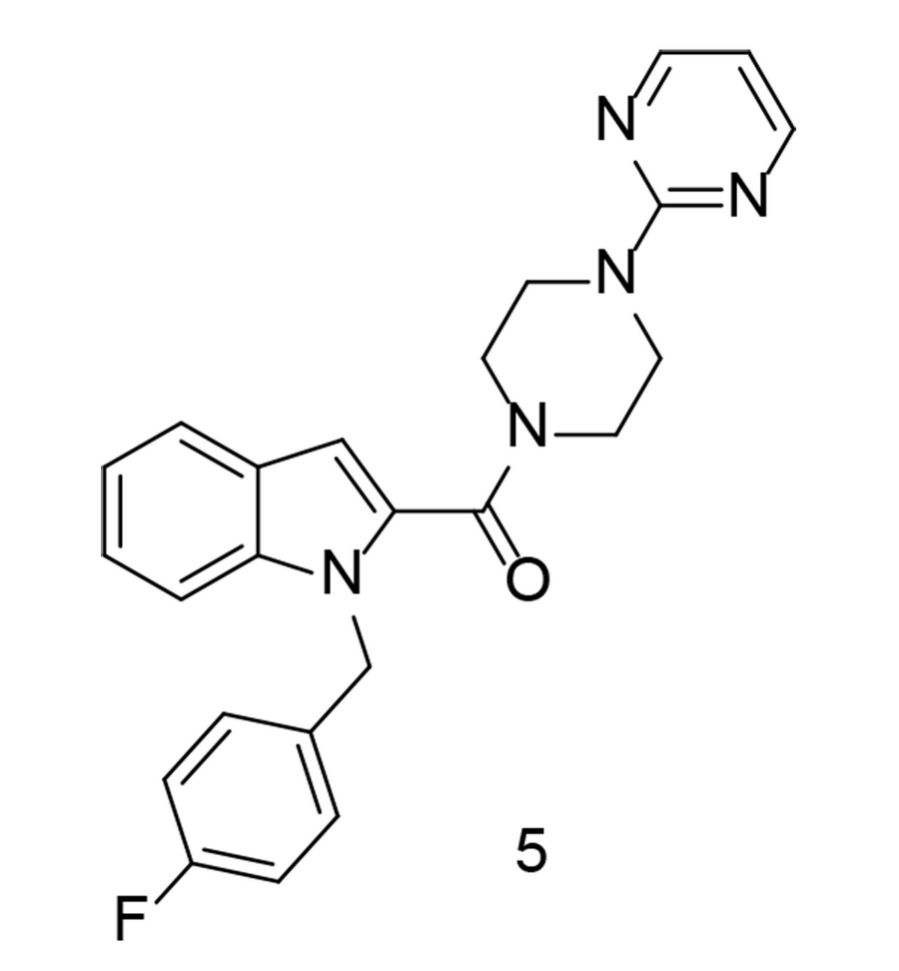
Indole-piperazine pyrimidine analogs

As a continuation of the research on heterocyclic compounds with COX-2 inhibitory activity, a study conducted by Wang et al. developed a new series of indole/indazole-piperazine pyrimidine analogs with neuroprotective properties (97). Among these compounds, compound 5 (Figure 8) exhibited the most promising cytoprotective efficacy against oxygen-glucose deprivation/reoxygenation-induced injury in BV2 microglial cells. Additionally, this compound significantly reduced the release of IL-1β, IL-6, TNF-α, and NO in LPS-induced BV2 cells. Moreover, compound 5 inhibited the release of TNF-α in a mouse model of neuroinflammation induced by LPS. As a potent inhibitor of both COX-2 and 5-LOX, compound 5 also suppressed M1 microglial phenotype polarization. Furthermore, it demonstrated substantial neuroprotective effects in rats, reducing infarct size and improving neurological impairment scores.

**Figure 9.**
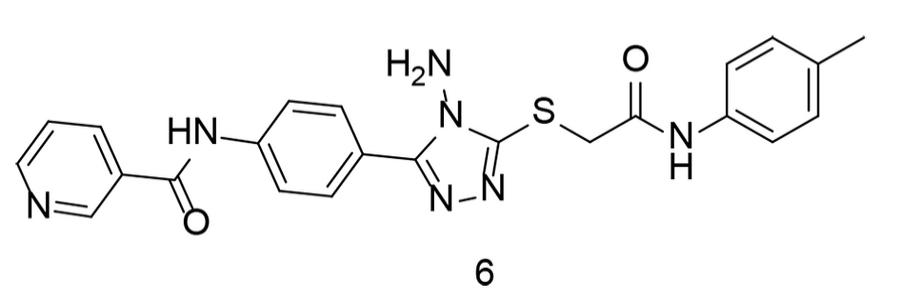
1,2,4-triazole-based derivatives as dual cyclooxygenases-2 (COX-2)/tumor necrosis factor-alpha (TNF-α) inhibitors

In terms of their ability to inhibit COX activity, oxadiazole-containing compounds were extensively studied. Two novel series of 1,3,4-oxadiazole and 1,2,4-triazole-based derivatives were synthesized and tested for their COX-2 inhibitory properties (98). In vitro studies showed that the new compounds were strong and selective COX-2 inhibitors. Their anti-inflammatory properties and protective effects against damage caused by harmful agents in macrophages were also evaluated. Many of these new compounds demonstrated excellent anti-inflammatory activity, surpassing that of celecoxib and diclofenac, particularly in inhibiting IL-6. Notably, compound 6 (Figure 9), a highly selective COX-2 inhibitor (IC_50_ = 0.04 μM, SI = 337.5), showed superior results in reducing ROS and NO production compared to celecoxib.

**Figure 10.**
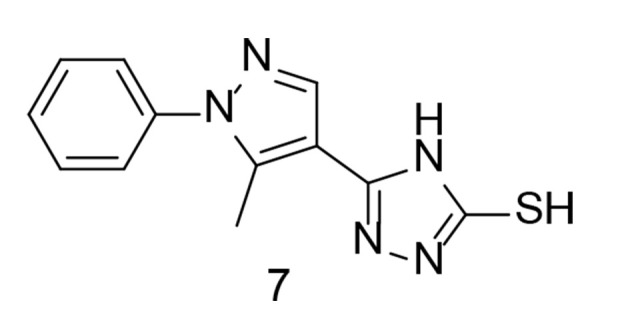
Pyrazole-triazole hybrid analogs as selective cyclooxygenases-2 (COX-2) inhibitors with cardioprotective effects

The cardiac adverse reactions associated with COX-2 selective medications were most severe for coxibs, leading to their removal from the market shortly after their introduction. Abdellatif et al. attempted to develop a new class of pyrazole compounds modified with a triazole ring as selective COX-2 inhibitors with cardiovascular protection (99). These compounds were synthesized and tested in vitro for their activity against COX-1 and COX-2. Compound 7 (Figure 10) exhibited the highest selectivity for the COX-2 enzyme (SI = 27.56) and proved to be the most effective anti-inflammatory agent. Cardiovascular profile evaluations showed comparable levels of heart-related biomarkers, along with inflammatory markers TNF-α and IL-6, similar to the positive control. The findings indicated that compound 7 had a superior cardiovascular profile compared to celecoxib. An ADME study was also conducted, and the new compounds demonstrated promising efficacy as emerging oral anti-inflammatory drugs.

**Figure 11.**
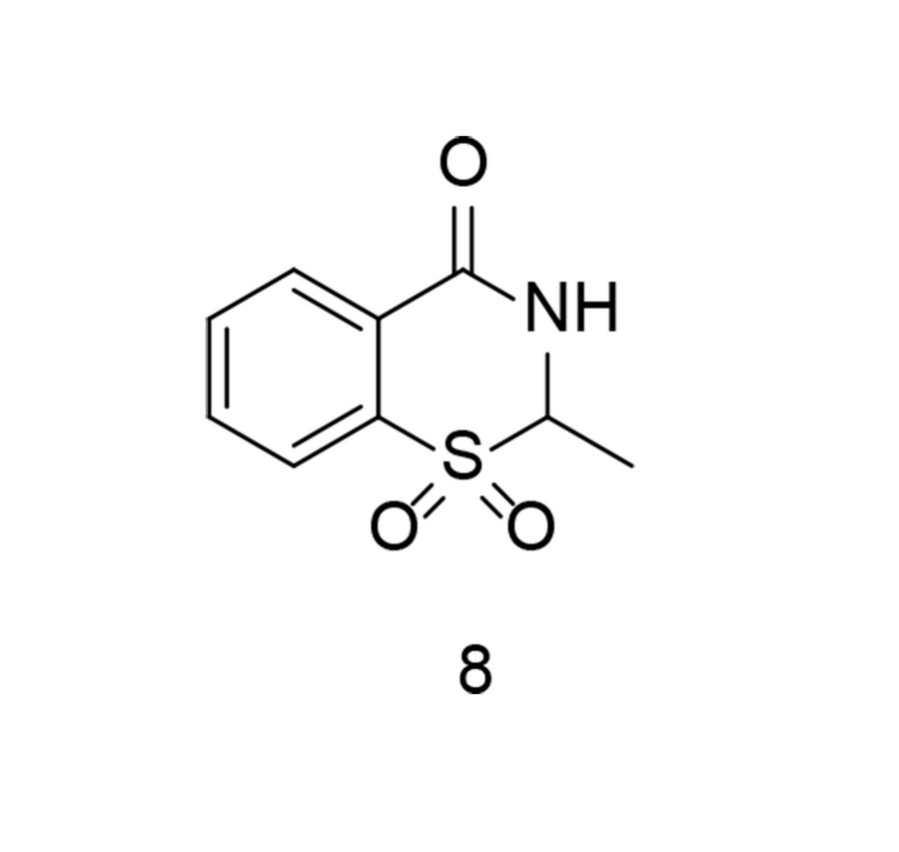
Sulfone-containing 1,3-benzothiazinone derivative as dual cyclooxygenases-2 (COX-2)/tumor necrosis factor-alpha (TNF-α) inhibitors

As previously discussed, the sulfonyl/sulfonamide motif is a characteristic pharmacophore group for selective COX-2 inhibition. On the other hand, some of the most prominent COX-2 inhibitors contain a benzothiazinone scaffold. Sulfone-containing 1,3-benzothiazinone derivatives represent novel scaffolds exhibiting anti-inflammatory properties with minimal toxicity. To identify an additional class of COX-2 inhibitors, a new series of 1,3-benzothiazinone derivatives was developed and synthesized. Several compounds demonstrated significant activity in inhibiting LPS-induced NO and TNF-α production, suppressing COX-2, and increasing IL-10 generation in RAW264.7 cells (100). None of the compounds showed noticeable cytotoxicity in vitro. Compound 8 (Figure 11) significantly reduced STAT3 and NF-κB phosphorylation in LPS-induced RAW264.7 cells. The compound also exhibited lower gastrointestinal toxicity compared to meloxicam. Furthermore, compound 8 reduced effusion in carrageenan-induced paw edema models of inflammation, while significantly lowering PGE2 concentrations.

**Figure 12.**
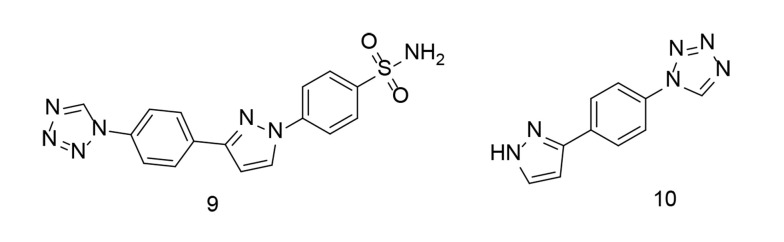
Tetrazole-bearing sulfonamide analogs as selective cyclooxygenases-2 (COX-2) inhibitors

To further investigate combined tetrazole-based sulfonamide analogs, a range of tetrazole-bearing compounds were synthesized and evaluated for their in vitro COX inhibitory action, as well as their anti-inflammatory activity by measuring IL-6 and TNF-α expression levels (101). Compound 9 (Figure 12) was found to be a highly effective and selective COX-2 inhibitor (COX-2 IC_50_ = 0.23 µM; COX-2 SI = 16.91). It was identified as the most potent derivative against TNF-α. Additionally, compound 10 (Figure 12) demonstrated broad-spectrum antibacterial activity against gram-positive cocci, gram-positive rods, and yeast fungi. Computational analysis revealed significant binding interactions for compound 9, including with the COX-2 pharmacophore (SO_2_NH_2_), generating two hydrogen bonds with His90 and Tyr355. Compound 9 fit perfectly within the COX-2 active site and exhibited the highest COX-2 SI of all the tested compounds.

**Figure 13.**
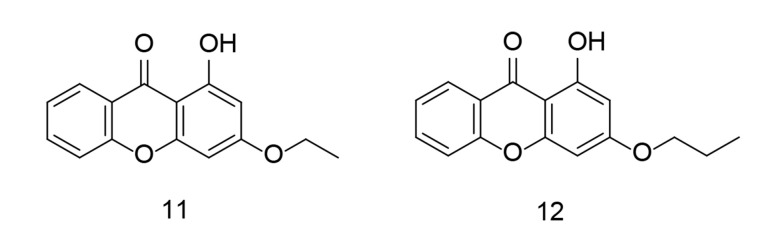
Substituted xanthone analogs as cyclooxygenases-2 (COX-2)/ tumor necrosis factor-alpha (TNF-α) inhibitors.

In 2023, combinatorial chemistry and computational studies helped researchers design xanthone moieties with high selectivity as potent COX-2 inhibitors (102) (Figure 13). The partially synthesized xanthone analogs were administered orally to carrageenan-induced paw edema rat models, and their efficacy in reducing inflammation was measured at specific time points. The results from in-silico, in-vitro, in-vivo, and analytical tests showed that hydroxyxanthone derivatives can effectively block COX-2. Furthermore, the newly developed xanthonic compounds were found to have a relatively low toxicity margin.

**Figure 14.**
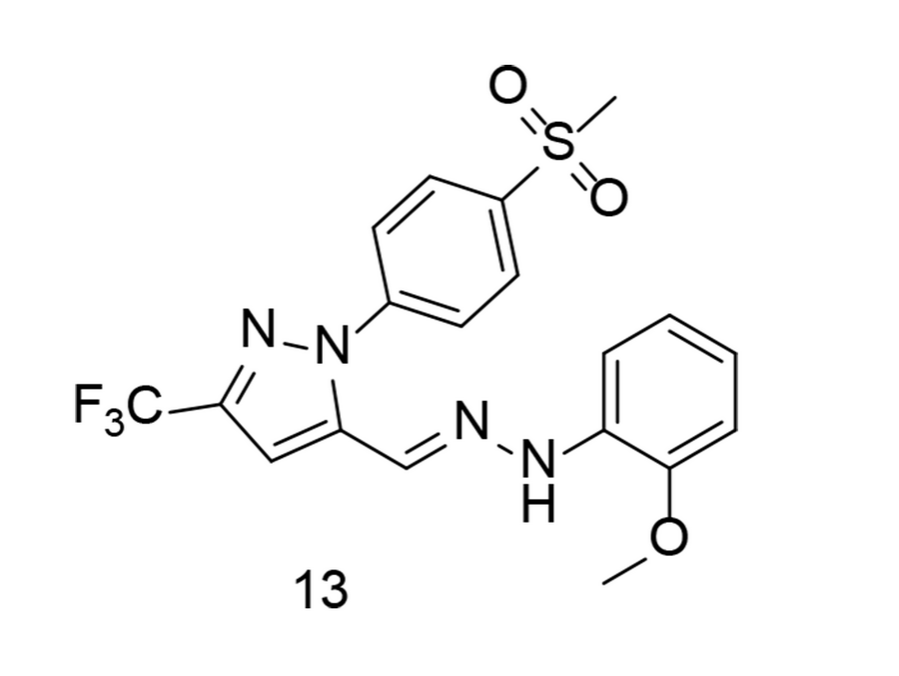
Natural anti-inflammatory ascidian metabolite 13 as cyclooxygenases-2 (COX-2)/ tumor necrosis factor-alpha (TNF-α) inhibitors

In a related study, Fang et al. applied a molecular hybridization technique in 2023 to introduce a new family of ascidian-celecoxib COX inhibitors using a hydrazone linker (103). The analogs underwent in silico analysis to evaluate their binding interactions with COX-2. The synthetic analogs were tested for their inhibitory properties across both COX enzymes, with compound 13 (Figure 14) showing effective, balanced inhibition. Compound 13 significantly inhibited the signal transduction pathway mediated by NF-κB in LPS-stimulated murine RAW264.7 macrophages, reducing the expression of pro-inflammatory TNF-α, COX-2, IL-1β, and IL-6, while also decreasing ROS and NO generation. Due to its high in vitro anti-inflammatory activity and lack of cytotoxicity, compound 13 appears to be a promising candidate for further research as a potential lead compound for targeting inflammation.

Using linkers to modify well-known COX-2 inhibitors has the potential to significantly improve specificity. Triazole analogs have been shown to exhibit slightly higher inhibitory efficacy and selectivity than certain other COX-2 inhibitors.

**Figure 15.**
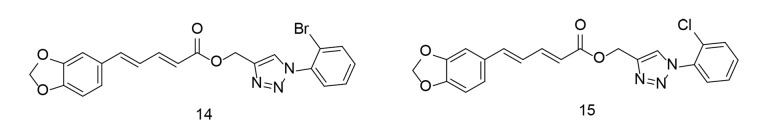
Piperine-based triazoles 14 and 15 as selective cyclooxygenases-2 (COX-2)/tumor necrosis factor-alpha (TNF-α) inhibitors

In 2015, Ali et al. developed a novel series of piperine-based triazoles with anti-inflammatory activity using a click chemistry approach. The most potent compounds were studied in vitro for their effects on TNF-α expression (104). Compounds 14 and 15 (Figure 15) were shown to significantly suppress inflammatory responses in vivo when compared to piperine and the conventional drug indomethacin, without causing stomach damage. Compounds 14 and 15 reduced TNF-α levels by 73.73% and 70.64%, respectively, as well as the protein expression of NF-κB, TNF-α, and COX-2, compared to indomethacin. Additionally, compound 14 demonstrated considerable analgesic efficacy of 54.09%, comparable to indomethacin.

**Figure 16.**
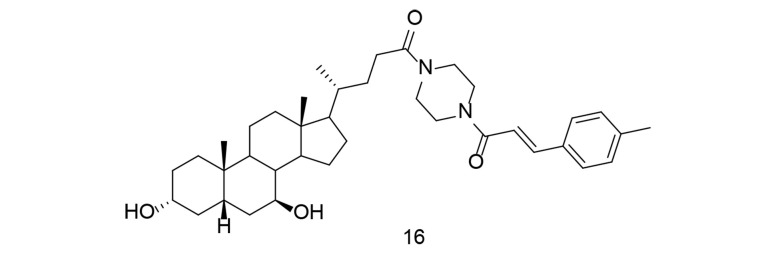
UDCA-cinnamic acid hybrid 16

With the rise of molecular hybridization as a promising approach in disease therapy, a new series of ursodeoxycholic acid-cinnamic acid hybrids was developed and synthesized. The anti-inflammatory potential of these compounds was evaluated by measuring their ability to inhibit LPS-induced NO production in RAW264.7 macrophages (105). Compound 16 (Figure 16) exhibited the highest inhibitory efficacy against NO (IC_50_ = 7.70 μM) and showed no notable toxicity. The study demonstrated that compound 16 significantly reduced TNF-α, IL-1β, IL-6, and PGE2 levels, while also down-regulating COX-2 expression. A preliminary mechanistic investigation revealed that compound 16 inhibited the Akt/NF-κB and MAPK signaling pathways, contributing to its anti-inflammatory effects. Additionally, compound 16 decreased inflammation in an in vivo mouse model of LPS-induced inflammatory disease. These findings suggest that cinnamic acid analogs, such as compound 16, hold promise as lead compounds for the development of anti-inflammatory drugs targeting both COX-2 and TNF-α.

**Figure 17.**
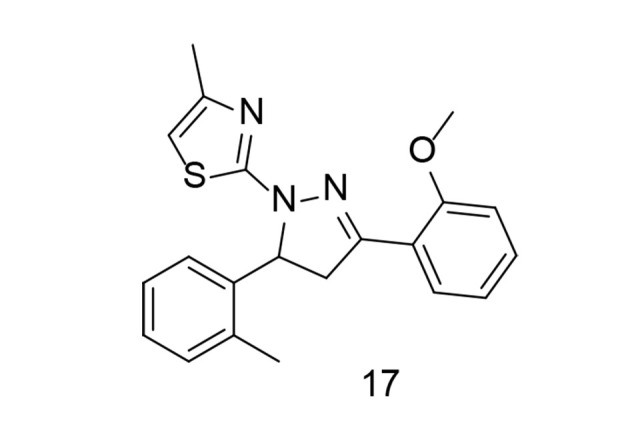
3,5-diaryl-4,5-dihydropyrazole-thiazole derivative 17

Systemic inflammation is a key factor in sepsis, one of the leading causes of mortality worldwide. It has been reported that 3,5-diaryl-4,5-dihydropyrazole and its derivatives exhibit anti-inflammatory and antibacterial properties. Using the pharmacophore combination approach, a research team led by Zhang incorporated a thiazole moiety into the dihydropyrazole skeleton to generate a novel series of methyl thiazole derivatives and explored their anti-inflammatory effects for sepsis treatment (106). Through initial SAR analysis based on their inhibitory effects against NO release in LPS-induced RAW264.7 cells, the optimal compound, 17 (Figure 17), demonstrated more potent anti-inflammatory activity than indomethacin and dexamethasone. Compound 17 effectively reduced IL-1β and TNF-α production, as well as the expression of iNOS and COX-2, by inhibiting the MAPK signaling pathway. Furthermore, in vivo administration of compound 17 significantly reduced the severity of LPS-induced sepsis in C57BL/6J mice, resulting in less damage to various organs. Overall, this research suggests that compound 17 may be a promising candidate for the treatment of sepsis.

Neuroinflammation refers to inflammation-related processes that occur within the cerebral cortex and spinal column, playing a role in the progression of various central nervous system (CNS) disorders, including Parkinson's disease (PD), Alzheimer's disease (AD), ischemia, and multiple sclerosis (MS). Microglial cells, which function like macrophages in the CNS, play a crucial role in the brain's innate defense mechanisms and are primarily responsible for inflammation-mediated neurodegeneration. Preventing microglial activation may reduce neuronal damage and degeneration under inflammatory conditions, making it a potential therapeutic strategy for CNS disorders.

**Figure 18.**
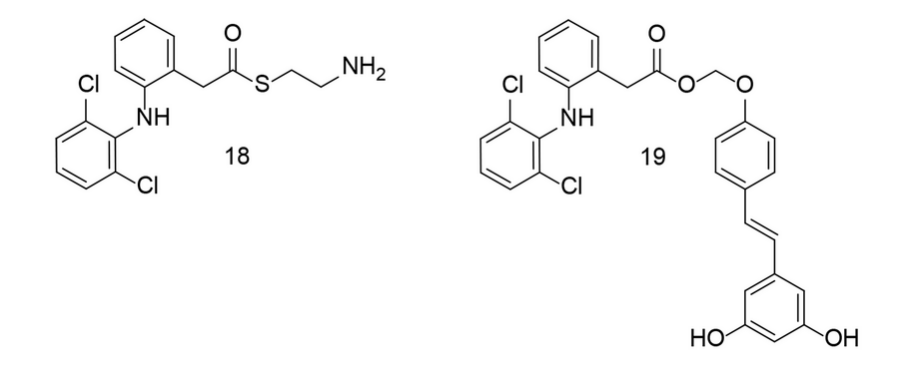
Aryl acetic acid conjugates 18 and 19 as dual cyclooxygenases-2 (COX-2)/tumor necrosis factor-alpha (TNF-α) inhibitors

Researchers synthesized several aryl acetic acid conjugates and evaluated their anti-inflammatory potential in LPS-stimulated BV-2 microglial cells and primary murine microglial cells (107). Compounds 18 and 19 (Figure 18) potently inhibited NO production while causing minimal cell damage. Compound 18 significantly reduced the levels of TNF-α, IL-6, COX-2, and iNOS in LPS-stimulated BV-2 microglial cells. Detailed molecular studies revealed that compound 18 notably inhibited MAPK phosphorylation and the subsequent activation of activator protein 1 (AP-1). Additionally, in a co-culture setup, compound 18 decreased the cytotoxicity of LPS-activated microglia on HT-22 neuroblastoma cells. Collectively, these experimental results suggest that compound 18 exerts a strong anti-neuroinflammatory effect by suppressing microglial activation.

**Figure 19.**
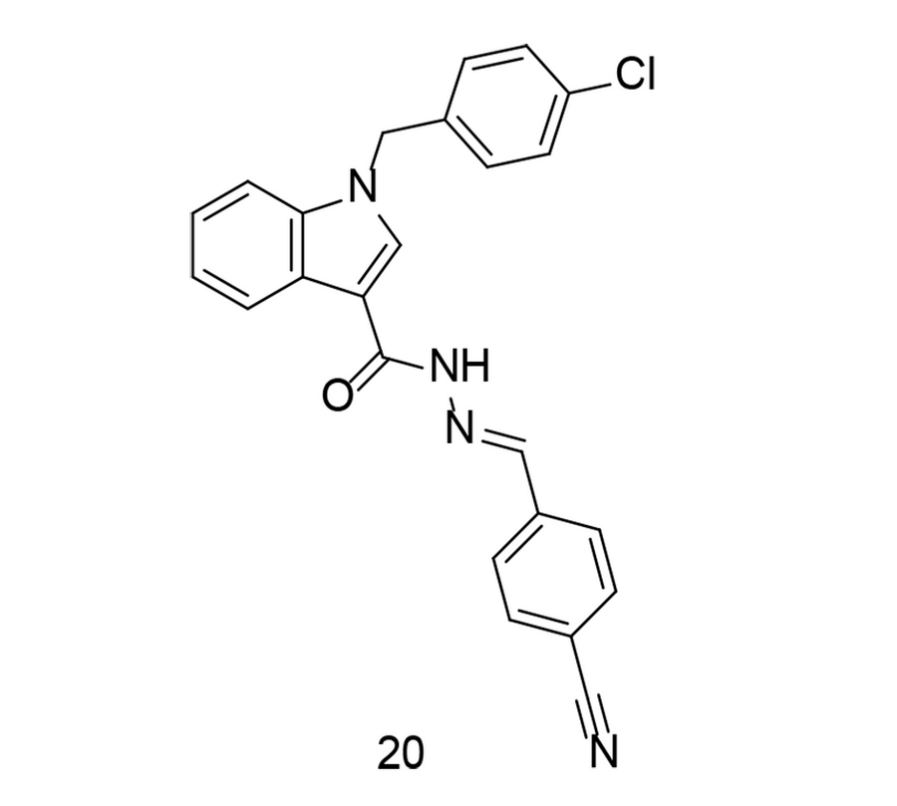
Indole-arylidene analog 20 as a dual cyclooxygenases-2 (COX-2)/tumor necrosis factor-alpha (TNF-α) inhibitor

In some cases, to develop a new series of COX-2 inhibitors with improved effects against TNF-α, compounds with balanced inhibitory activity against both COX isozymes were preferred over selective COX-2 inhibitors. In a study conducted by Ju et al., a novel series of substituted indole arylidene analogs incorporating structural motifs from ascidians, with balanced COX-1/COX-2 inhibition, was introduced (108). These newly synthesized analogs possess a molecular structure similar to anthranilic acid analogs that inhibit COX. The novel analogs were tested for their inhibitory activity against COX-1 and COX-2 enzymes, and compound 20 (Figure 19) was further evaluated for its in vitro anti-inflammatory capabilities. Compound 20 inhibited the production of pro-inflammatory factors such as TNF-α, COX-2, and IL-6 in LPS-stimulated murine RAW264.7 macrophages. Additionally, suppression of PGE2, NO, and ROS was observed, along with inhibition of NF-κB and IκBα phosphorylation. The study concluded that compound 20 acts as a mixed inhibitor of COX-1/COX-2, reducing ROS production and suppressing NF-κB, while also inhibiting the production of TNF-α, iNOS, IL-6, and COX-2.

**Figure 20.**
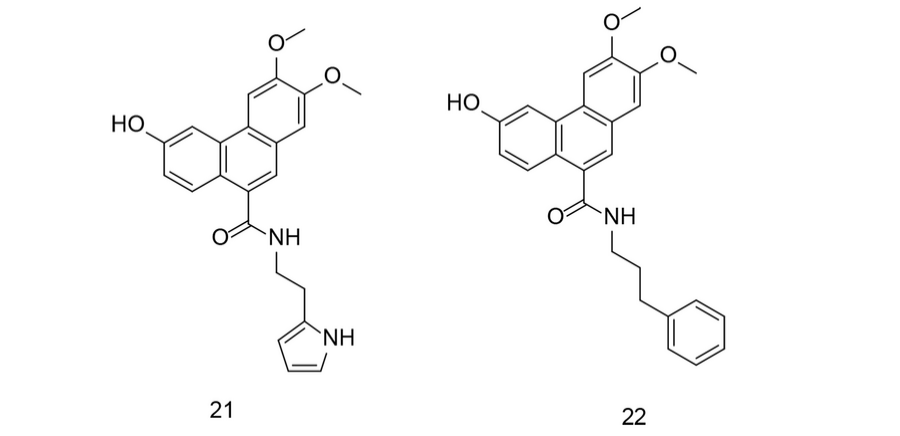
Novel 9-phenanthranilamide derivatives 21-22

Oxidative stress refers to an imbalance between free radicals and antioxidants in the body. Free radicals are highly reactive molecules containing an unpaired electron, making them unstable. They can damage cells, proteins, and DNA through a process called oxidation. Normally, the body can manage a certain amount of free radicals and maintain a balance with antioxidants. However, when there is an excess of free radicals or a deficiency in antioxidants, oxidative stress occurs. This can lead to cellular and tissue damage, which has been associated with various health conditions, including inflammation, aging, neurodegenerative diseases, cardiovascular diseases, and cancer. After a stroke, the brain faces challenges in recovery due to excessive inflammation and stress. In a study by Du et al., several new 9-phenanthranilamide derivatives were synthesized and evaluated for their anti-inflammatory and antioxidant activities (109). The newly discovered compounds significantly reduced LPS-induced production of IL-6, IL-1β, and TNF-α in microglial cells. Furthermore, compounds 21 and 22 (Figure 20) effectively suppressed NO generation and provided dose-dependent protection to PC12 neuronal cells triggered by H_2_O_2_. Both compounds suppressed the NF-κB signaling pathway and reduced LPS-induced inflammatory mediators such as iNOS and COX-2. Further investigation revealed that compound 22 had a significant neuroprotective effect in rats with middle cerebral artery blockage, reducing infarct volume and improving neurological deficit scores. Overall, the findings suggest that compound 22 could be a promising candidate for stroke therapy.

**Figure 21.**
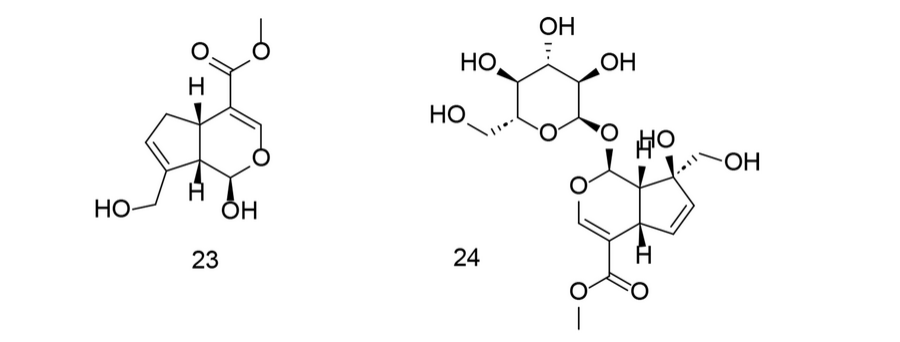
Aspirin GEP ester analogs 23, and 24

As previously emphasized, aspirin and other nonsteroidal anti-inflammatory drugs (NSAIDs) are effective cancer chemopreventive agents. On the other hand, genipin is a natural cross-linking agent that inhibits lipid peroxidation and NO generation. Genipin has been shown to protect hippocampal neurons from the harmful effects of Alzheimer's amyloid beta protein. To target COX-2 using a combination of NSAIDs and genipin (compound 23), an innovative class of aspirin GEP ester counterparts was created through automated screening and synthesized via an esterification reaction (110). The newly synthesized compounds were tested in collagen-induced arthritis models using sequential enzymatic assays, western blot analysis, and pathological observations. Compound 23 (Figure 21) exhibited greater binding affinity with COX-1 and the lowest calculated free energy value with COX-2, compared to other ligands developed by combining NSAIDs with GEP. Compound 24 (Figure 21) demonstrated dose-dependent COX-2 inhibition and selectivity in successive enzymatic assays and reduced COX-2 expression in the peripheral blood cells of CIA rats. Additionally, compound 24 decreased swelling in the hind paw, alleviated arthritis symptoms, and helped prevent tissue damage around the joints and in the lymph nodes of arthritis-afflicted rats.

Butenolides are a class of chemical compounds characterized by a four-membered lactone ring, a double bond, and a ketone group. Butenolides, such as γ-butyrolactone, are commonly used as precursors in the synthesis of various chemical compounds. They have been researched for their role as signaling molecules in plants, regulating processes such as seed germination, flowering, and defense mechanisms. Butenolides have also been studied for their pharmacological properties, including potential anticancer, antibacterial, and insecticidal activities. Researchers continue to investigate the synthesis and biological activities of butenolides to fully realize their potential in a wide range of scientific and industrial fields.

**Figure 22.**
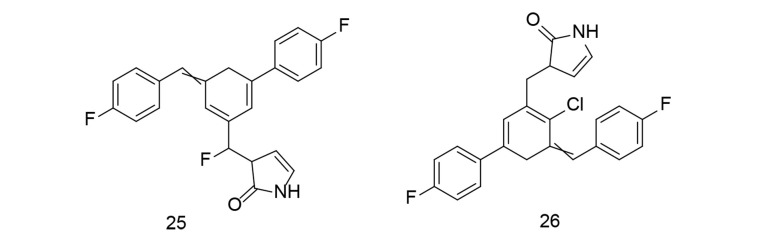
Novel butenolide-based benzyl pyrrolones 25-26

Ali et al. developed a library of new butenolide-based benzyl pyrrolones and conducted in-silico molecular docking experiments against TNF-α (111). All synthesized compounds were evaluated for in vivo anti-inflammatory efficacy using a carrageenan-induced paw edema model. Substances with strong anti-inflammatory activity were further tested for in vitro TNF-α production. Compounds 25 and 26 (Figure 22) demonstrated substantial inhibition of 76.22% and 71.47%, respectively, which was slightly lower than the reference, indomethacin. Compounds 25 and 26 reduced TNF-α levels by 65.03% and 60.90%, respectively, compared to indomethacin's 68.84% suppression, indicating that indomethacin can effectively inhibit TNF-α activity. Compound 25 also exhibited considerable analgesic efficacy at 60.04%, which was comparable to that of indomethacin. Additionally, compounds 25 and 26 were examined for their effects on the protein expression of NF-κB and COX-2 in liver tissues. These compounds provided better protection for the stomach and reduced inflammation compared to indomethacin.

Alzheimer's disease is a progressive neurological disorder that leads to cognitive decline and memory loss. It is the most common cause of dementia, which is characterized by a severe decline in thinking and memory, affecting a person's ability to perform daily tasks. Alzheimer's disease is marked by abnormal brain structures called β-amyloid plaques and tau protein tangles. β-amyloid plaques are extracellular deposits of a protein fragment called β-amyloid, while tau protein tangles are intracellular aggregations of hyperphosphorylated tau protein. These abnormalities lead to neural dysfunction, synaptic loss, and ultimately, neuronal death. Symptoms of AD include memory loss, particularly recent memory, as well as difficulties with language, mental agility, and visuospatial skills. As the disease progresses, individuals may experience personality changes, mood swings, and behavioral issues. The primary risk factor for AD is advanced age, with most cases occurring in people over 65.

There is currently no cure for AD, and available therapies are primarily aimed at alleviating symptoms and slowing the progression of the disease. Cholinesterase inhibitors (e.g., donepezil, rivastigmine) and memantine (an NMDA receptor antagonist) may help improve cognitive symptoms and enhance the quality of life for some patients. Additionally, non-pharmacological treatments such as cognitive stimulation, physical activity, and social engagement may offer further benefits. Research into AD is ongoing, with efforts focused on understanding the underlying causes, identifying promising biomarkers for early diagnosis, developing more effective treatments, and ultimately discovering a cure or preventative measures.

As discussed, AD is thought to result from β-amyloid (Aβ)-induced proteopathy and immunopathology. Monomeric Aβ is derived from amyloid precursor protein and aggregates into oligomers and fibrils, which disrupt neuronal membrane integrity and cause cell damage. Aβ is neurotoxic and synaptotoxic, and it may also induce neuroinflammation by activating microglial cells. Previous research demonstrated that furosemide, an anthranilate-based medication, can downregulate the proinflammatory microglial M1 phenotype. Wang and his team developed new drugs based on furosemide to target both Aβ accumulation and brain inflammation, which are key factors contributing to AD (112).

**Figure 23.**
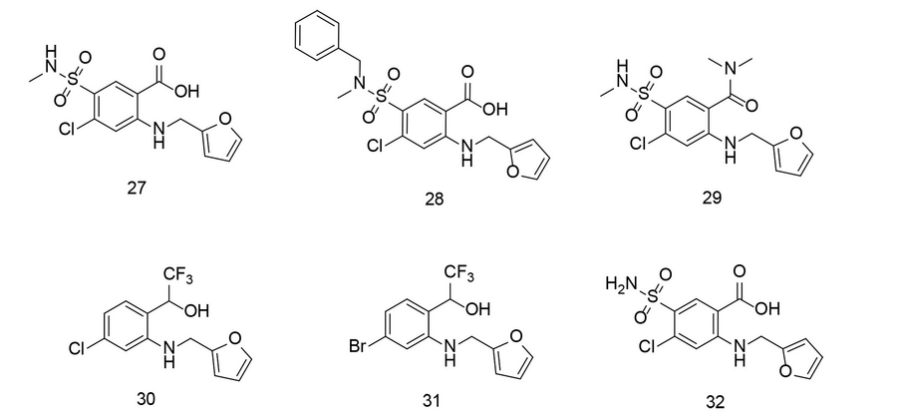
Furosemide analogs 27 - 31 and the parent compound furosemide 32

Compounds 27 - 29 (Figure 23) reduced Aβ oligomerization, while compounds 30 and 31 (Figure 23) inhibited Aβ fibrillization. Compounds 28 and 31 also suppressed TNF-α, IL-6, and NO production, reduced COX-2 and iNOS expression, and enhanced microglial phagocytosis, demonstrating dual efficacy against both Aβ aggregation and neuroinflammation.

**Figure 24.**
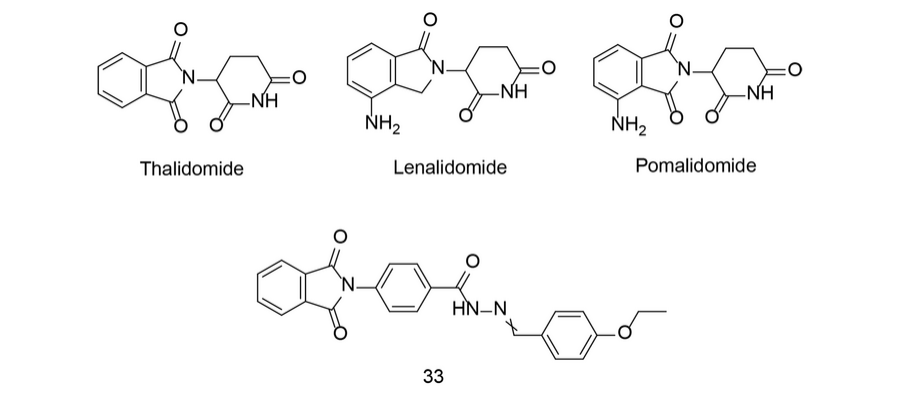
N-aryl phthalimide 33, derived from thalidomide

Thalidomide (Figure 24) is a medication originally developed in the late 1950s as a sedative to relieve morning sickness in pregnant women. However, it was later found to cause severe birth defects when taken during pregnancy, leading to one of the biggest pharmaceutical disasters in history. By the early 1960s, reports linked thalidomide use during pregnancy to a high incidence of babies born with limb deformities, such as phocomelia. The discovery of these devastating birth defects prompted regulatory agencies to withdraw thalidomide from the market in many countries. Subsequent investigations revealed that thalidomide interferes with the development of limbs and other structures in the fetus when taken during a critical period of pregnancy.

Despite its tragic history, thalidomide has found new uses in modern medicine. It has been repurposed to treat certain conditions, including leprosy and multiple myeloma, a type of cancer affecting plasma cells in the bone marrow. Thalidomide and its derivatives, such as lenalidomide and pomalidomide (Figure 24), are now used in combination with other medications to treat these conditions, with strict controls in place to prevent use during pregnancy and minimize the risk of birth defects.

It is also worth noting that thalidomide inhibits COX-2 and can significantly reduce TNF-α production. In a study conducted by Bhat et al., N-arylphthalimides derived from thalidomide, modified by the addition of hydrophobic groups, were evaluated for their anti-inflammatory effects, with compound 33 emerging as a potential anti-inflammatory agent. In a mouse model of lung inflammation, treatment with compound 33 significantly reduced TNF-α, IL-6, IL-17, and NF-κB-positive cell populations while increasing Foxp3 and IκBα-positive cell populations in whole blood and pleural fluid (113). Compound 33 therapy also reduced inflammation-associated proteins (COX-2, TNF-α, NF-κB, IL-6) while increasing anti-inflammatory cytokines (IL-4 and IL-10). Additionally, histological examination confirmed the potent anti-inflammatory actions of compound 33. Thus, the N-arylphthalimide derivative 33 was shown to be a strong anti-inflammatory drug in the carrageenan-induced lung inflammation model.

P38α, also known as MAPK14, is a member of the MAPK family, specifically the p38 subfamily. Mitogen-activated protein kinases are serine/threonine protein kinases that play crucial roles in a variety of biological processes, including cell proliferation, differentiation, apoptosis, and stress responses. P38α/MAPK14 is activated in response to stress stimuli such as cytokines, UV irradiation, heat shock, and osmotic stress. Once activated, P38α/MAPK14 phosphorylates downstream substrates that regulate cellular responses, including transcription factors and protein kinases.

P38α/MAPK14 regulates the production of inflammation-related substances like cytokines, chemokines, and other mediators, thereby modulating inflammatory reactions. It is involved in both chronic and acute inflammatory processes. Activation of P38α/MAPK14 can lead to cellular senescence and apoptosis, both of which are critical for maintaining tissue homeostasis and removing damaged or undesirable cells. Additionally, P38α/MAPK14 plays a key role in modulating immune cell activities, including proliferation, cytokine production, and migration. It is implicated in immune responses to infections and autoimmune diseases. Furthermore, P38α/MAPK14 is involved in various cellular differentiation processes, such as myogenesis, adipogenesis, and hematopoiesis.

**Figure 25.**
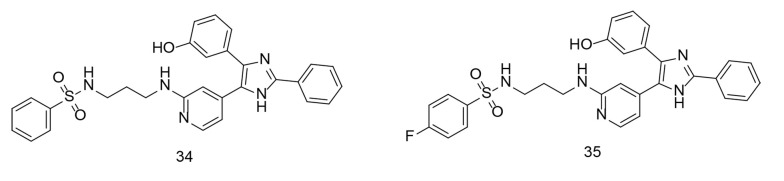
Compounds 34 and 35 as multi-acting agents against proinflammatory cytokines

Overall, P38α/MAPK14 plays diverse and critical roles in cellular physiology and pathology, making it an important target for therapeutic intervention in various diseases, including inflammation-related disorders and cancer. P38α/MAPK14 is an intracellular signaling regulator that controls the production of inflammatory cytokines such as TNF-α, IL-1, IL-6, and IL-1β. These cytokines trigger the creation of inflammatory proteins, including iNOS, NF-κB, and COX-2. The study applied pharmacological repurposing strategies to shift B-RAF V600E imidazole-5-yl pyridine inhibitors toward the inhibition of P38α kinase. Another study used P38α kinase inhibitors to develop a pharmacophore model, which demonstrated their strong binding affinity at the P38α active site. Compounds 34 and 35 (Figure 25) were found to be the most effective inhibitors of P38α kinase, with IC_50_ values of 47 nM and 45 nM, respectively. Compound 35 significantly reduced the production of IL-6, IL-1β, and TNF-α in LPS-induced RAW 264.7 macrophages, with IC_50_ values of 78.03 nM, 17.6 µM, and 82.15 nM, respectively. The anti-inflammatory efficacy of these compounds was evaluated by measuring the reduction in PGE2 and NO production in LPS-stimulated RAW 264.7 macrophages. Compound 35 effectively inhibited PGE2 and NO production, with IC_50_ values of 0.29 µM and 0.61 µM, respectively. Molecular dynamics simulations of inhibitor 35 demonstrated its conformational stability at the P38α kinase binding site.

Inflammatory bowel diseases (IBD) are chronic, idiopathic inflammatory conditions affecting the gastrointestinal tract. The two main types of IBD are Crohn's disease and ulcerative colitis. While both conditions involve chronic inflammation of the digestive tract, they differ in the location and nature of the inflammation. Crohn's disease can affect any part of the digestive tract, from the mouth to the anus, although it most commonly affects the end of the small intestine (ileum) and the beginning of the large intestine (colon). Inflammation in Crohn's disease is patchy, meaning some areas are affected while others remain healthy. Symptoms of Crohn's disease may include stomach pain, weight loss, fatigue, diarrhea, and nutrient deficiencies. Ulcerative colitis primarily affects the large intestine and rectum. In this condition, inflammation usually starts in the rectum and can extend throughout the colon. Symptoms of ulcerative colitis may include bloody stools, abdominal pain, rectal bleeding, urgency to defecate, and fatigue.

**Figure 26.**
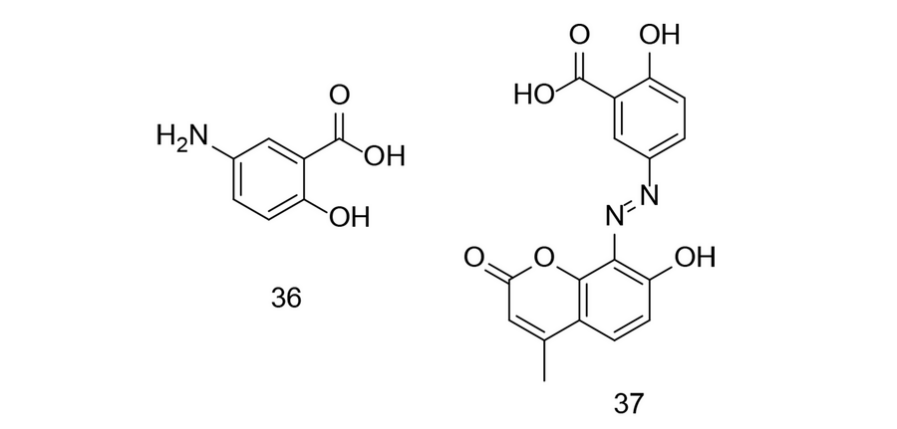
Mesalamine 36 and multiple sclerosis (MS)-CU analog 37

IBD has no known medical cure, and treatment focuses on alleviating symptoms and maintaining remission. Research has shown that persistent inflammation in ulcerative colitis is a significant risk factor for cancer development. Mesalamine, a COX inhibitor, is commonly used to manage IBD symptoms. Numerous studies have demonstrated mesalamine's chemopreventive effect against cancer, primarily due to its anti-inflammatory properties. Cyclooxygenases-2 inhibition may help prevent colorectal cancer (CRC) by promoting apoptosis, reducing cell proliferation, and regulating angiogenesis. Recent research investigated the synthesis of mesalamine linked to coumarin via a diazo group (114). Molecular docking experiments were conducted with four target proteins (COX-2, MMP-9, TNF-α, and MPO) to explore the interaction between mesalamine (compound 36, Figure 26) and its coumarin derivative, MS-CU. MS-CU (compound 37, Figure 26) showed stronger binding affinity for all tested proteins (MMP-9, MPO, COX-2, and TNF-α) compared to mesalamine alone. Molecular dynamics simulations demonstrated that both MS and MS-CU formed stable complexes with the proteins in a water environment. Additionally, MS-CU (compound 37) was found to prevent colonic injury in a rat model of ulcerative colitis induced by acetic acid.

**Figure 27.**
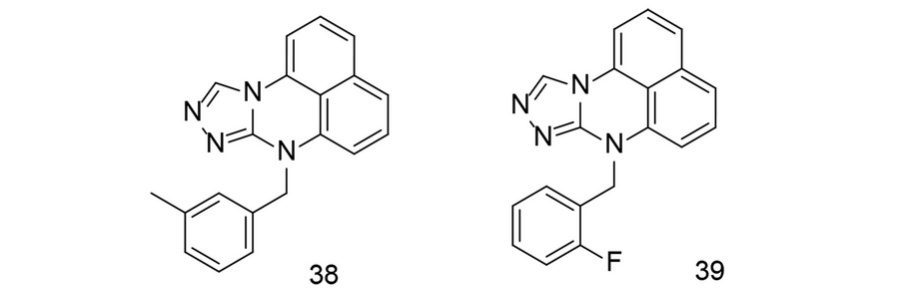
Pyrimidine analogs 38, and 39

Over the past several years, extensive studies have been conducted to develop COX-2 inhibitors using tetracyclic main scaffolds. One study focused on the design, synthesis, and anti-inflammatory effects of a class of pyrimidine derivatives that include triazole (115). The anti-inflammatory properties of these synthesized pyrimidine analogs were examined using an LPS-stimulated inflammation model. Compounds 38 and 39 (Figure 27) reduced TNF-α and IL-6 levels in RAW264.7 cells. The anti-inflammatory efficacy of compounds 38 and 39 was further tested in vivo using a xylene-induced ear inflammation model. Compound 38 showed the strongest anti-inflammatory activity, reducing inflammation by 49.26%. This activity was higher than that of the reference drug, ibuprofen, but slightly lower than that of indomethacin. To better understand the inhibitory effects, LPS-induced NF-κB activation and MAPK phosphorylation were analyzed. Western blotting experiments revealed that compound 39 reduced NF-κB activation and MAPK phosphorylation in a dose-dependent manner. Additionally, a docking study of compound 39 at the COX-2 binding site suggested that its mechanism may be similar to naproxen. The impact of compound 39 on COX-2 antibodies significantly decreased COX-2 activity.

Ginsenosides are a class of bioactive compounds found in the roots, stems, and leaves of plants in the genus Panax, particularly Panax ginseng (Asian ginseng) and Panax quinquefolius (American ginseng). These compounds are responsible for many of the health benefits associated with ginseng, a popular herbal remedy used in traditional Chinese medicine for thousands of years. Ginsenosides are saponins, a type of glycoside compound characterized by a steroid or triterpene core structure with one or more sugar molecules attached. They are known to exert various pharmacological effects on the body, including adaptogenic properties, meaning they may help the body adapt to stress and improve resilience.

Ginsenosides also possess antioxidant properties, which can help protect cells from damage caused by free radicals and oxidative stress. This antioxidant activity may contribute to their anti-aging effects and ability to support cardiovascular health. Reports have shown that some naturally occurring ginsenosides exert antioxidative effects by activating antioxidant enzymes. For instance, treatment with ginsenosides was found to decrease NO and PGE2 levels in LPS-challenged RAW264.7 cells and mouse livers. Furthermore, the biological effects of ginsenosides were associated with the down-regulation of iNOS, COX-2, and NF-κB activity in both in vitro and in vivo studies.

Some research suggests that ginsenosides can modulate the immune system, enhancing immune function and potentially reducing the risk of infections. They have also been investigated for their potential neuroprotective effects, including the ability to improve cognitive function, enhance memory, and protect against neurodegenerative diseases. Ginsenosides may exert anti-inflammatory effects, which could be beneficial for conditions characterized by chronic inflammation, such as arthritis and inflammatory bowel disease.

**Figure 28.**
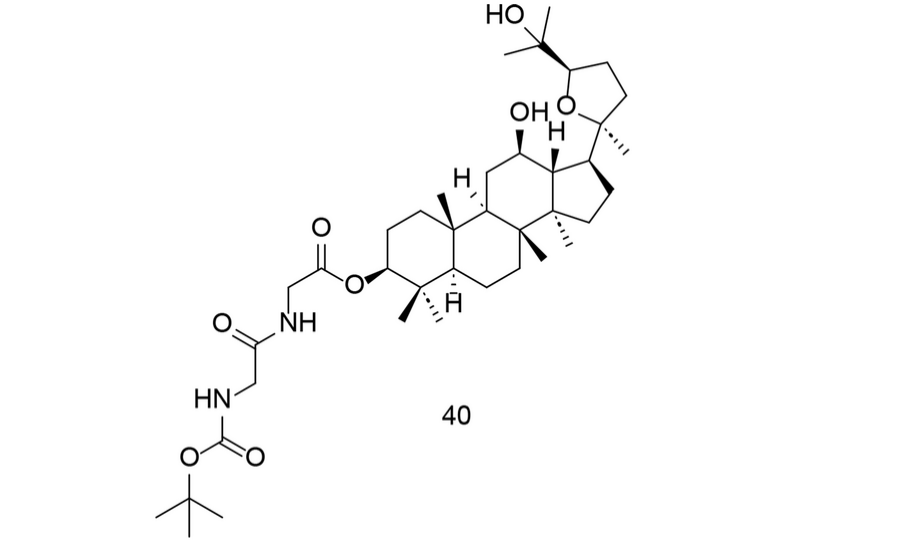
Ocotillol-type sapogenins (OTS) ginsenoside metabolite 40

Research into the pharmacological properties and potential therapeutic applications of ginsenosides is ongoing, and they continue to be a subject of interest in both traditional and modern medicine. Ocotillol-type sapogenins (OTS) are the primary ginsenoside byproducts in human liver tissue. To more effectively exploit OTS and its derived compounds as anti-inflammatory agents, researchers created numerous new 3-amino acid OTS derivatives and tested their anti-inflammatory potential in vitro (116). N-Boc-protected neutral aliphatic amino acid derivative of 24R-OTS 40 (Figure 28) showed the strongest efficacy in vitro, outperforming even hydrocortisone sodium succinate. Compound 40 effectively inhibited LPS-induced elevation of TNF-α, IL-6, iNOS, and COX-2 through the NF-κB and MAPK pathways. This suggests that OTS derivatives could be promising anti-inflammatory drugs that require further preclinical testing.

**Figure 29.**
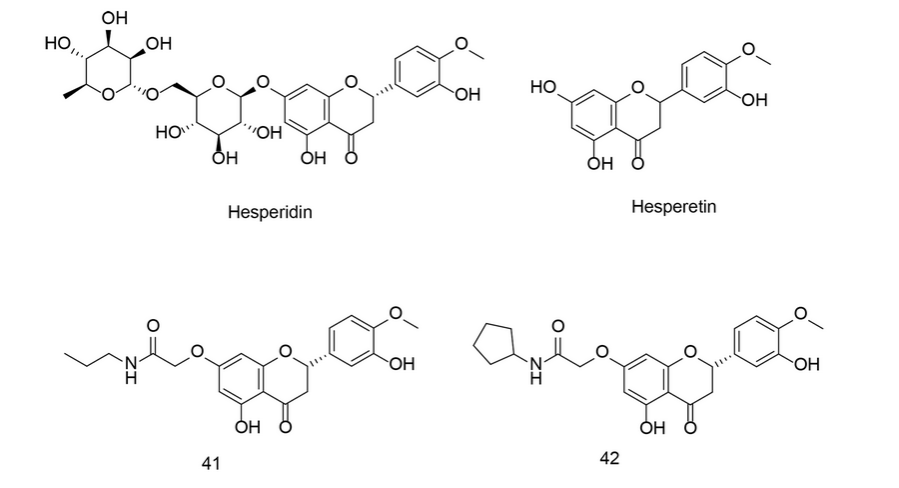
7-O-amide hesperetin derivatives 41, and 42

Hesperetin (Figure 29) is a flavonoid, a plant-derived antioxidant compound found in citrus fruits such as oranges, lemons, and grapefruits, as well as in various other fruits and vegetables. It belongs to the flavanone class and is closely related to the flavonoid hesperidin (Figure 29). Hesperetin has demonstrated antioxidant and anti-inflammatory properties, along with cardiovascular health benefits. Research indicates that it may help lower blood pressure, cholesterol levels, and improve blood vessel function, potentially reducing the risk of coronary artery disease.

Hesperetin has also been investigated for its neuroprotective effects, specifically its potential to guard against neurodegenerative disorders. Moreover, hesperetin has shown antiproliferative activity, modulating multiple signaling pathways in cancer cells related to growth, metastasis, and apoptosis. Recently, hesperetin has gained attention for its potential anti-COVID-19 activity, with several studies suggesting that it could be used not only as an anti-COVID-19 agent but also as a preventative measure against cancer by inhibiting key signaling pathways, particularly COX-2 inhibition.

To develop novel anti-inflammatory drugs, a series of 7-O-amide hesperetin derivatives were synthesized and evaluated for anti-inflammatory activity in RAW264.7 cells. All of the newly identified compounds demonstrated inhibitory effects on LPS-induced NO production (117). Among these, compounds 41 and 42 (Figure 29), which possess hydrophobic side chains, exhibited the most potent NO inhibitory activity and showed a stronger inhibitory effect on the production of TNF-α, IL-1β, and IL-6 compared to indomethacin and celecoxib. The SAR analysis indicated that the 7-O-amide unit was situated in a medium-sized hydrophobic pocket of the receptor. Compound 41 also inhibited the expression of COX-2 and iNOS via the NF-κB pathway.

**Figure 30.**
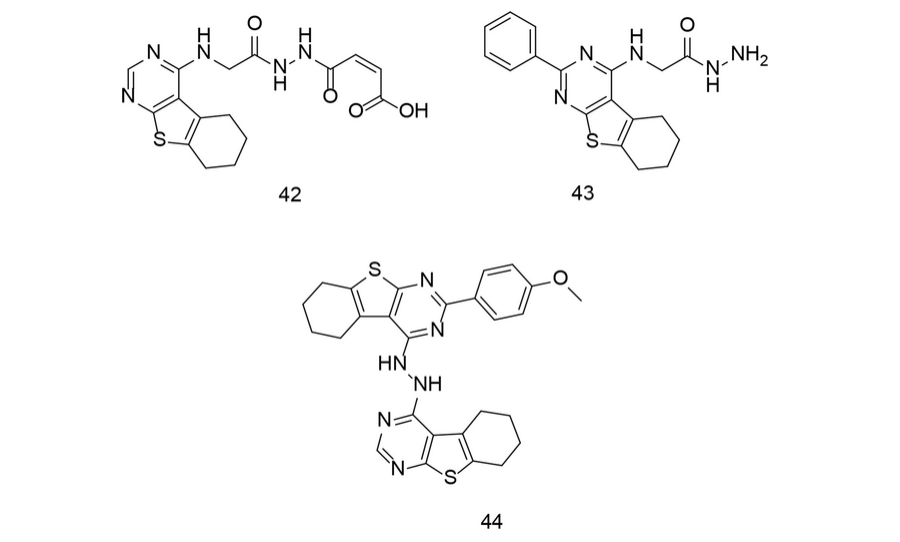
Thieno[2,3-d]pyrimidine derivatives 42-44.

As discussed in previous reviews by our research team (91, 92), developing ligands with dual inhibitory activities for COX-2 and LOX enzymes is a novel approach to introducing new anticancer agents. In a study conducted by Abdelkhalek et al., a new series of thieno[2,3-d]pyrimidine derivatives were designed and synthesized to develop multitarget-directed ligands as dual 15-LOX/COX-2 inhibitors (118). The effectiveness of these compounds against inflammatory cytokines, such as ROS, NO, TNF-α, and IL-6, was assessed in RAW 264.7 macrophages stimulated with LPS. Compound 42 (Figure 30) inhibited 15-LOX more effectively than nordihydroguaiaretic acid, with an IC50 of 1.17 μM compared to 1.28 μM for the latter. The phenyl-substituted acetohydrazide 43 (Figure 30) exhibited the best COX-2 selectivity, although it was lower than that of celecoxib. Compound 42 showed the strongest 15-LOX inhibitory activity and had five-fold higher COX-2 selectivity than diclofenac. It was more effective in reducing NO production than celecoxib and diclofenac, with equivalent TNF-α inhibition to diclofenac. Similarly, compound 43 reduced IL-6 and TNF-α levels more effectively than diclofenac.

As an extension of the "miraculous slingshot" strategy for anti-inflammatory medication, new thienopyrimidine monomers/heterodimers were introduced as dual COX-2/15-LOX inhibitors. New tetrahydrobenzo[4,5]thieno[2,3-d]pyrimidine monomers/heterodimers were discovered to be dual COX-2/15-LOX inhibitors with strong antioxidant activity (119). The synthetic substances were tested in various in vitro biological experiments. The heterodimer 44 (Figure 30) was the most effective and selective compound in the study, with IC50 values of 65 nM and 1.86 μM for COX-2 and 15-LOX, respectively. Additionally, the newly synthesized compounds significantly reduced LPS-induced production of ROS and pro-inflammatory cytokines (IL-6, TNF-α, and NO) in RAW 264.7 macrophages. The heterodimer suppressed ROS (IC_50_ = 18.79 μM) and IL-6 (IC_50_ = 4.15 μM) production more effectively than celecoxib and diclofenac.

**Figure 31.**
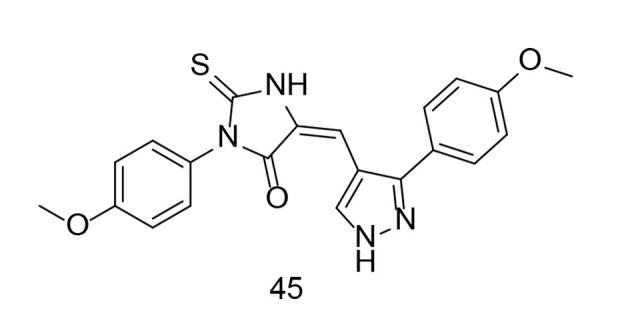
Pyrazole-based analogs 45

As discussed earlier in this paper, celecoxib is a 1H-pyrazole analog with selective COX-2 inhibitory activity, substituted by 4-sulfamoyl phenyl, trifluoromethyl, and p-tolyl groups at positions 1, 3, and 5, respectively. This specific structure confers celecoxib its selective inhibitory action against COX-2, distinguishing it from non-selective NSAIDs. The precise arrangement of atoms and functional groups in celecoxib's structure is crucial for its pharmacological activity and therapeutic effects in managing pain and inflammatory conditions. In an experiment conducted by Wang and colleagues, a new group of potential COX-2, TNF-α, and IL-1β inhibitors featuring a pyrazole-based scaffold were developed (120). Among these newly developed derivatives, compound 45 (Figure 31) exhibited the most significant anti-inflammatory, antipyretic, and prolonged analgesic effects, along with mutual inhibitory action on all tested enzymes.

**Figure 32.**
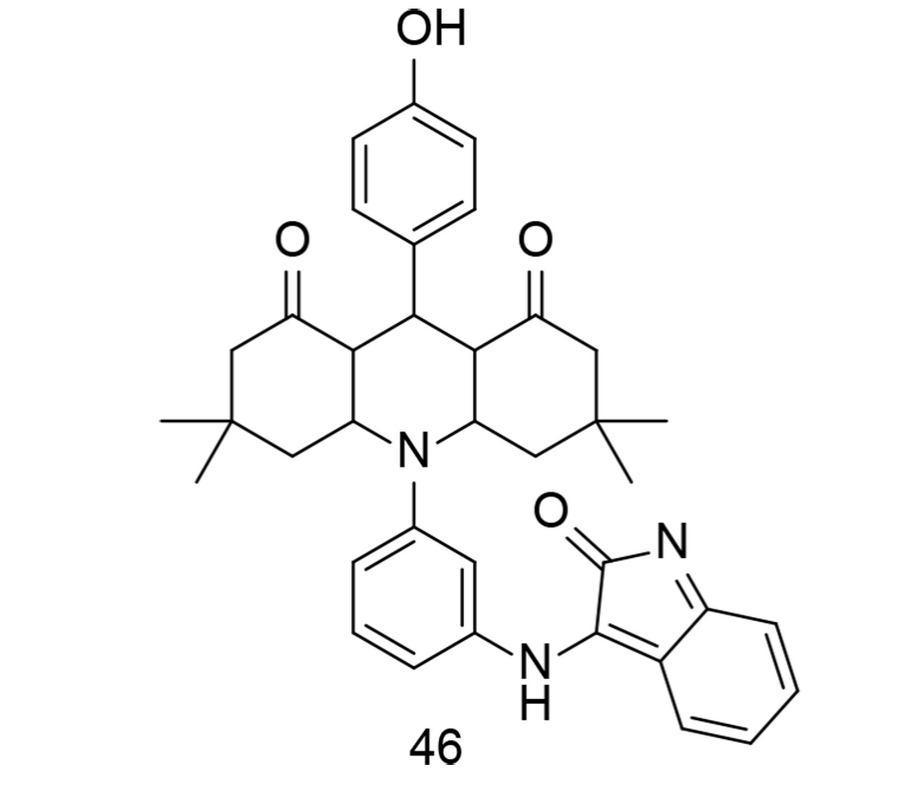
Isatinimino-acridinedione core 46

Isatin is a valuable heterocyclic organic compound derived from indole, found in certain plants, and can also be produced synthetically. Its presence in nature has inspired interest in studying its biological activities and potential pharmacological applications. Isatin derivatives have been explored in medicinal chemistry for a wide range of biological activities, including antimicrobial, antiviral, anticancer, and anti-inflammatory properties. Modifications to the isatin core structure can yield compounds with enhanced pharmacological properties and improved target specificity. A successful synthesis of an isatinimino acridinedione moiety with impactful anti-inflammatory responses, achieved through the inhibition of the in vivo NF-κB and COX-2 signal transduction pathways, was reported by Periyasami et al. (121). These newly synthesized analogs were created by combining hydrophilic, fluorescent, and COX-2 inhibitory components into a single compound. The isatinimino-acridine-dione core was synthesized through a straightforward synthetic process. In vivo biological studies demonstrated the compounds' excellent anti-inflammatory properties and efficacy. The produced isatinimino analog, compound 46 (Figure 32), inhibited the transcription of iNOS, COX-2, and NF-κB proteins, as well as the production of PGE2, NO, TNF-α, IL-1β, and IL-6 levels induced by carrageenan.

**Figure 33.**
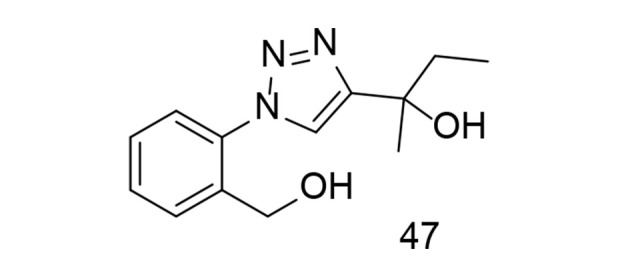
Phenyl-1H-1,2,3-triazole analog 47

Diaryl triazole analogs have been shown to induce dual COX-2/TNF-α inhibitory activities in previous studies (122, 123). In a study focusing on triazole analogs as selective COX-2 inhibitors, a comparative molecular field investigation was conducted to explore the SAR, and pharmacophores with effective anti-inflammatory properties were identified. The anti-inflammatory effect was evaluated through the transcription of TNF-α-induced COX-2. Ultimately, it was found that the use of analog 47 (Figure 33) caused significant changes in the expression levels of TNF-α-induced COX-2 (124).

**Figure 34.**
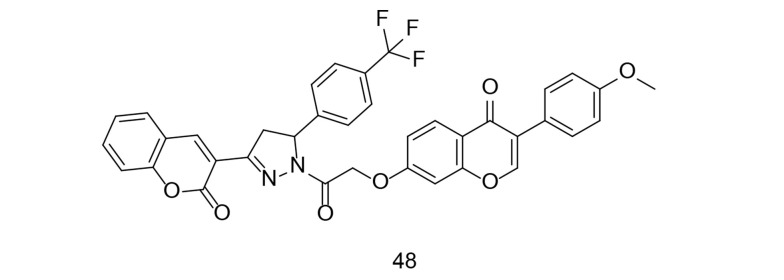
Phenyl-pyrazoline-coumarin analog 48

Coumarins, also known as benzopyrans, are versatile compounds found naturally in plants and have been used in herbal remedies for a long time. They are present in various plants, such as tonka beans, cinnamon, and sweet clover, and exhibit a wide range of biological activities. Some coumarins have photoprotective properties, making them useful in sunscreen formulations due to their ability to absorb UV radiation. Among their most notable activities are anticoagulant, anticancer, antihypertensive, and anti-inflammatory effects. Coumarins have gained significant interest for their ability to block COX-2, thereby reducing inflammation. Chen et al. developed and synthesized a series of phenyl-pyrazoline-coumarin derivatives (125). All substances were tested for their anti-inflammatory effects by measuring the reduction of LPS-induced IL-6 production. Compound 48 (Figure 34) showed the strongest anti-inflammatory effect by reducing IL-6, TNF-α, and NO production. This compound effectively inhibited iNOS, COX-2, IL-6, TNF-α, and NO synthesis through the NF-κB/MAPK signaling pathway.

**Figure 35.**
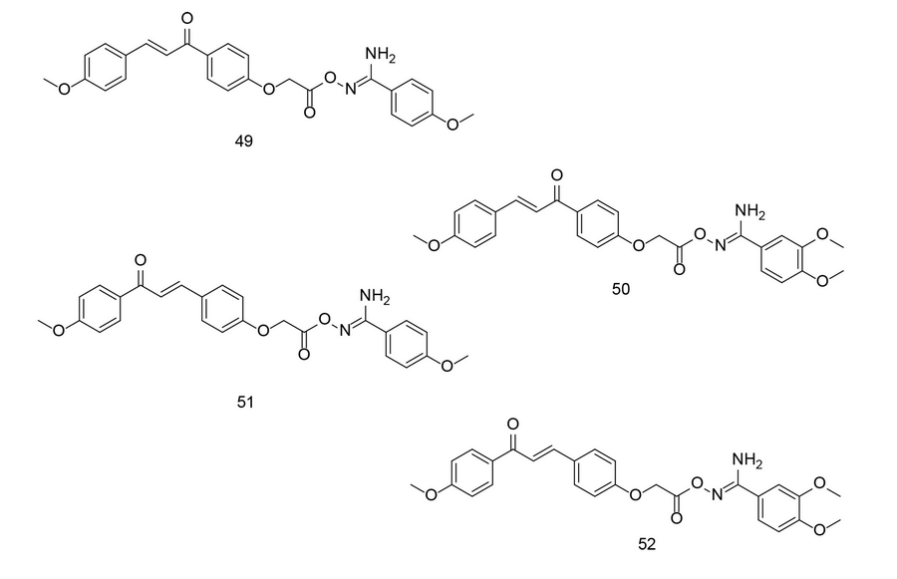
Chalcone/aryl carboximidamide hybrids

Chalcones, precursors of flavonoids and isoflavonoids, can act on various drug targets such as COX-2 and TNF-α. Chalcones have shown potential effectiveness against both drug-susceptible and drug-resistant cancers, making them promising candidates for the development of new antitumor agents (94, 126). The key pharmacophore of chalcone analogs is their core, an α, β-unsaturated carbonyl-containing scaffold, which preferentially inhibits COX-2. Our research group has been involved in the development of chalcone derivatives as potential selective COX-2 inhibitors for the past few decades (127, 128), (76, 77, 87, 129-132). In a study conducted by Ibrahim et al., two new sets of chalcone/aryl carboximidamide hybrids were designed and evaluated as novel pharmacological agents against PGE2 (133). Among the newly developed compounds, 49, 50, 51, and 52 (Figure 35) were found to be the most effective inhibitors of PGE2 and NO production induced by LPS. Moreover, the aforementioned compounds demonstrated significant edema inhibition compared to indomethacin and celecoxib. Additionally, compound 49 showed good COX-2 inhibitory activity.

## 7. Discussion

The COX enzymes are among the most well-researched and understood mammalian oxygenases. COX enzymes, with their two distinct but related active sites, catalyze the bis-dioxygenation and subsequent reduction of AA to PG. Nonsteroidal anti-inflammatory drugs have been used in therapeutic settings since ancient Egypt, long before COX was recognized as a biological target. Based on the COX-2 hypothesis, selective COX-2 inhibitors were developed to reduce the gastrointestinal toxicity commonly associated with NSAIDs. Cyclooxygenases-2 plays a significant role in the pathogenesis of inflammation, which has been linked to various serious medical conditions such as osteoarthritis, rheumatoid arthritis, cancer, and neurodegeneration. Consequently, COX-2 remains an important but challenging target for pharmaceutical development.

In much the same way that our understanding of COX-inhibitor interactions has required the integration of multiple complementary experimental techniques, the future use of COX-2 as a pharmaceutical target may necessitate a range of synthetic approaches. The effectiveness of COX-2 inhibitors highlights the critical role of eicosanoids in various disorders. Fully harnessing the potential of the COX pathway could serve as a model for an emerging paradigm of multimodal approaches in pharmaceutical research. Given the enzyme's importance in cancer pathophysiology, there has been a growing trend over the past three decades to develop and produce innovative selective COX-2 inhibitors as tumor chemopreventive agents.

Cyclooxygenases-2 inhibitors are gaining attention for their potential influence on the expression of TNF-α genes. Tumor necrosis factor-alpha has been implicated in cancer through its association with chronic inflammation, leading to numerous studies on this topic. A comprehensive investigation is required to identify drugs that can effectively inhibit both COX-2 and TNF-α. To develop novel medications that target both COX-2 and TNF-α, it is essential to first understand the SAR of COX-2 inhibitors that can simultaneously inhibit TNF-α. The SAR of selective COX-2 inhibitors has been extensively studied. Cyclooxygenases-2 inhibitors exhibit diverse chemical structures. Unlike traditional NSAIDs, these enzyme inhibitors generally lack a carboxylic group, which influences their binding to COX-2 through an alternative site within the enzyme, without forming a salt bridge in the enzyme’s hydrophobic channel.

Selective COX-2 inhibitors typically consist of a 1,2-diaryl substitution on a central heterocyclic, carbocyclic, or linear component, featuring a sulfonyl, sulfonamide, azido, methanesulfonamide, or tetrazole group on one of the aryl rings. This group functions as the pharmacophore, which is crucial for COX-2 selectivity. These structural components are essential for the selective inhibition of COX-2 activity. Numerous studies have been conducted to identify the precise SAR of newly developed COX-2 inhibitors. For example, in a series of 2,4,5-triaryl imidazoles with a SO_2_CH_3_ pharmacophore at the para position of the C-4 phenyl ring, it was found that the type of substituent on the C-2 phenyl ring significantly affects COX-2 inhibitory potency and selectivity. The order of selectivity was: OH > F > OMe > H, Me > NHCOMe > Cl. In addition to traditional tricyclic COX-2 inhibitors like coxibs, non-traditional structures also exist, such as non-tricyclic inhibitors. These compounds lack a cyclic core and instead feature acyclic core systems with α,β-unsaturated ketone molecules. A vicinal diaryl structure is necessary to achieve selective COX-2 inhibition; otherwise, the compound will preferentially inhibit COX-1.

We need to determine the correlation between the structure of COX-2 inhibitors and their ability to suppress TNF-α. Generally, drugs with stronger COX-2 inhibitory properties are more effective at inhibiting TNF-α gene transcription. Designing a compound with potent COX-2 inhibitory efficacy may facilitate the reduction of TNF-α gene expression. More research is required to fully understand how the chemical structures of COX-2 inhibitors affect their ability to decrease TNF-α activity. In some cases, compounds with less selective inhibitory effects on COX-2, or those closer in structure to non-selective NSAIDs, may be more effective at inhibiting TNF-α gene expression. It is noteworthy that in many instances, the effects of these compounds on TNF-α align with their inhibitory effects on NO and NF-κB. This is an important area that requires further exploration.

In summary, regarding the SAR of COX-2 inhibitors, key structural features include aromatic rings capable of hydrophobic interactions, the incorporation of sulfonamide moieties, and the substitution of aromatic rings with groups of appropriate sizes and shapes. Understanding these structural aspects aids in designing more potent COX-2/TNF-α inhibitors with potential therapeutic benefits. This work is one of the few articles investigating the relationship between COX-2 inhibition and the expression of the gene encoding TNF-α. In recent years, numerous studies have explored the role of selective COX-2 inhibitors as cancer chemopreventive agents, summarizing the relationship between their chemical structures and their activity as COX-2 inhibitors. Additionally, various studies have been published regarding compounds that inhibit inflammation and suppress the activity of inflammatory cytokines like TNF-α. However, articles investigating the SAR of selective COX-2 inhibitors and their suppressive effects on inflammatory cytokines, particularly TNF-α, remain limited. This work aims to contribute to advancing knowledge in this area.

### 7.1. Conclusions

This publication summarizes novel chemical compounds that exhibit dual COX-2/TNF-α inhibitory activity. Enhanced COX-2 inhibition opens up new opportunities for developing drugs with measurable anti-TNF-α efficacy and low side effects. The properties of pharmacophores can lead to the discovery of COX-2 inhibitor scaffolds that inhibit TNF-α, iNOS, and NF-κB, much like finding the perfect recipe. In conclusion, dual COX-2/TNF-α suppression represents an innovative approach for developing new drugs for pain relief and cancer therapy, which should be pursued with strong emphasis.

## Data Availability

The dataset presented in the study is available on request from the corresponding author during submission or after publication.
